# Identification of QTL, QTL-by-environment interactions, and their candidate genes for resistance HG Type 0 and HG Type 1.2.3.5.7 in soybean using 3VmrMLM

**DOI:** 10.3389/fpls.2023.1177345

**Published:** 2023-04-21

**Authors:** Haipeng Jiang, Suchen Lv, Changjun Zhou, Shuo Qu, Fang Liu, Haowen Sun, Xue Zhao, Yingpeng Han

**Affiliations:** ^1^ Key Laboratory of Soybean Biology in Chinese Ministry of Education (Key Laboratory of Soybean Biology and Breeding/Genetics of Chinese Agriculture Ministry), Northeast Agricultural University, Harbin, China; ^2^ Daqing Branch, Heilongjiang Academy of Agricultural Science, Daqing, China

**Keywords:** soybean cyst nematode, GWAS, 3VmrMLM, QTL, candidate genes

## Abstract

**Introduction:**

Soybean cyst nematode (SCN, Heterodera glycines Ichinohe) is an important disease affecting soybean yield in the world. Potential SCN-related QTLs and QTL-by-environment interactions (QEIs) have been used in SCN-resistant breeding.

**Methods:**

In this study, a compressed variance component mixed model, 3VmrMLM, in genome-wide association studies was used to detect QTLs and QEIs for resistance to SCN HG Type 0 and HG Type 1.2.3.5.7 in 156 different soybean cultivars materials.

**Results and discussion:**

The results showed that 53 QTLs were detected in single environment analysis; 36 QTLs and 9 QEIs were detected in multi-environment analysis. Based on the statistical screening of the obtained QTLs, we obtained 10 novel QTLs and one QEI which were different from the previous studies. Based on previous studies, we identified 101 known genes around the significant/suggested QTLs and QEIs. Furthermore, used the transcriptome data of SCN-resistant (Dongnong L-10) and SCN-susceptible (Suinong 14) cultivars, 10 candidate genes related to SCN resistance were identified and verified by Quantitative real time polymerase chain reaction (qRT-PCR) analysis. Haplotype difference analysis showed that Glyma.03G005600 was associated with SCN HG Type 0 and HG Type 1.2.3.5.7 resistance and had a haplotype beneficial to multi-SCN-race resistance. These results provide a new idea for accelerating SCN disease resistance breeding.

## 1 Introduction

Soybean cyst nematode (SCN, *Heterodera glycines* Ichinohe) is an important disease in soybean production in the world, which is characterized by wide distribution, serious harm, a wide host range, many ways of transmission, and the long survival time of the dormant body (cyst) (Cook et al., 2012). It is a soil-borne disease that is extremely difficult to control. In general, it can reduce soybean yield by 30%–50%; however, in severe cases, they can cause complete loss (Cook et al., 2012). The annual loss of production caused by SCN is conservatively estimated at 2 billion US dollars (Wrather and Koenning, 2006). Rational rotation, biological control, and chemicals used can control SCN to a certain extent, but the most economical, safe, and effective way to control SCN was still the breeding and utilization of disease-resistant varieties (Winter et al., 2006; Kadam et al., 2016). Soybean plants ‘PI88788’, ‘PI209332’, ‘PI548402’, and ‘Peking’ (PI 548402) were the main sources of resistance in commercial varieties (Mitchum, 2016). The widespread and lasting use of these varieties has led to the gradual overcoming of this resistance by SCN populations in the field (Colgrove and Niblack, 2008). Therefore, it was particularly important to extensively screen and identify new varieties of resistance to SCN in soybean germplasm resources.

In China, SCN HG Type 0 and HG Type 1.2.3.5.7 were widely prevalent in the east and north, resulting in a serious loss of soybean yield. Among them, SCN HG Type 0 was the most widely distributed, and SCN HG Type 1.2.3.5.7 was the most harmful (Han et al., 2017). Soybean resistance to SCN was a complex quantitative character controlled by a few major genes and multiple minor genes (Concibido et al., 2004; Wang et al., 2004). So far, researchers have located more than 300 quantitative trait loci (QTLs) (www.soybase.org) on 20 chromosomes, most of which are minor QTLs, of which two major QTLs *rhg1* (Peking-type *rhg1-a* and PI88788-type *rhg1-b*) and *Rhg4* (*GmSHMT08*) have been widely used in SCN resistance breeding (Cook et al., 2012; Liu et al., 2012). Among them, the SCN resistance of *rhg1* was determined by the copy number of the three genes (Glyma.18G022500, Glyma.18G022500, and Glyma.18G022700); the more the copy number, the stronger the resistance, rather than the previously predicted leucine-rich repeat receptor-like kinases (LRR-RLKs). SCN resistance at *Rhg4* was caused by two point mutations in *GmSHMT* (Glyma.08G108900). Two non-synonymous mutations changed the regulation of serine hydroxymethyl-transferase, causing the programmed death of syncytial cells or triggering resistance signaling pathways, resulting in the death of pathogenic nematodes (Liu et al., 2012). The PI88788-type requires at least 5.6 copies of *rhg1-b* (Cook et al., 2012; Patil et al., 2019), whereas the Peking-type requires *rhg1-a* and *Rhg4* for SCN resistance (Patil et al., 2019). The resistance mechanism of soybean to SCN was complicated. In addition to *rhg1* and *Rhg4*, some minor SCN resistance genes have been reported (Vaghchhipawala et al., 2004; Klink et al., 2007; Kandoth et al., 2011; Mazarei et al., 2011; Lin et al., 2013).

The genome-wide association study (GWAS) was a strategy based on high-throughput genotyping technology, through association analysis, to take all single-nucleotide polymorphisms (SNPs) in the whole genome of a species as molecular genetic markers, to carry out control analysis or correlation analysis with target traits, to estimate the effects of detection sites through different models, and then to statistically test the estimated effects, so as to find the genetic variation of complex traits (Harold et al., 2009). Since the association analysis of agronomic traits was carried out in rice for the first time, the GWAS has been used to identify quantitative trait nucleotide (QTN) and candidate genes that control hundreds of plant quantitative or qualitative traits, including soybean, rice, corn, wheat, and other crops (Hanif et al., 2021; Rakotoson et al., 2021; Wang et al., 2021; Yuan et al., 2021). The application of the GWAS was inseparable from utility tools and model software. Plant traits were mostly controlled by a small number of major genes and a large number of minor genes; thus, the combination of multiphenotype, multienvironment, and multilocus models in the GWAS could make the mapping of trait loci more accurate (Zhang et al., 2019). Recently, a new model has been proposed, a compressed variance component mixed model, 3VmrMLM (Li et al., 2022). This model was a multimarker whole genome scanning model. This model not only has high QTL detection ability and sensitivity but also can detect QTL-by-environmental interaction (QEI) and QTL-by-QTL interaction (QQI). Previously, we identified 19 association signals significantly associated with resistance to two SCN HG Types (HG Type 0 and HG Type 1.2.3.5.7) using 35,760 SNPs (Han et al., 2015). However, there was a lack of GWAS studies on HG Type 0 and HG Type 1.2.3.5.7 resistance using the 3VmrMLM model.

The aims of this study were to use the 3VmrMLM model to analyze 23,150 SNPs and 156 different soybean cultivars collected from China, to identify new QTLs and QEI of soybean resistance to HG Type 0 and HG Type 1.2.3.5.7, to further understand the genetic structure of soybean resistance to HG Type 0 and HG Type 1.2.3.5.7 and to predict candidate genes that might regulate soybean SCN resistance in the linked genomic region with peak SNPs.

## 2 Materials and methods

### 2.1 Evaluation of soybean germplasm resistance to soybean cyst nematode HG type 0 and HG Type 1.2.3.5.7

**Figure 1:**
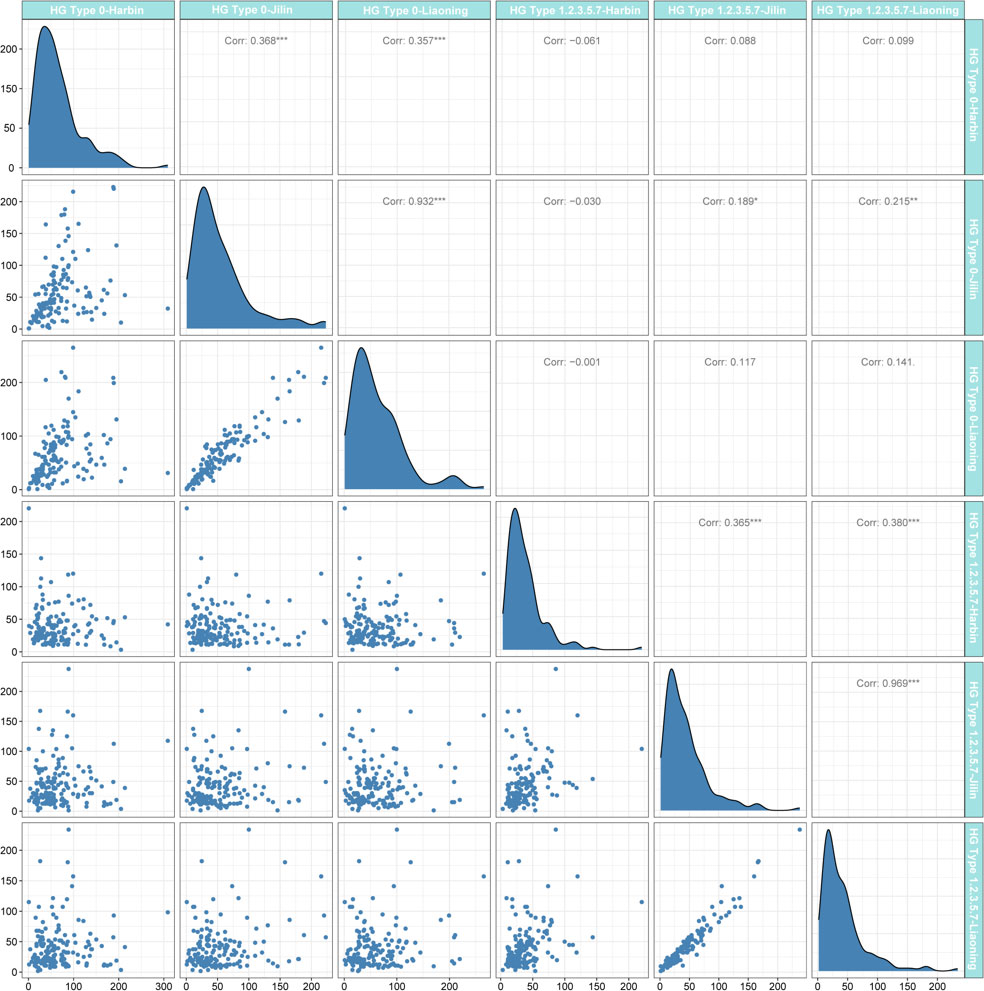
Distribution of female index (FI) after soybean cyst nematode (SCN) stress in three environments and the Pearson coefficient. The regression statistics between the two environments are below the diagonal, and the diagonal histogram shows the distribution of the FI in each environment, and correlation coefficients are above the diagonal (positive numbers represent positive correlations; negative numbers represent negative correlations).

In this study, 156 soybean cultivars were planted at Harbin (117°17′E, 33°18′N), Liaoning (41°48′N, 123°25′E), and Jilin (124°82′E, 43°50′N) in 2021 (**Table S1**). A random complete block design (RCBD) was used with three replicates of 10 seedlings each. The resistance of 156 soybean cultivars to SCN HG type 0 and HG Type 1.2.3.5.7 was identified, and the nematode inoculation method were performed following Jiang et al. (2021). The cysts and females of the tested accessions were collected and measured 30 days after the accessions were inoculated. The female index (FI) was calculated as FI = (number of cysts and females on detected plant)/(average number of cysts and females on ‘Lee 68’) × 100. FI > 10 and FI < 10 were designated ‘+’ and ‘−’, respectively (Golden et al., 1970). Pearson correlation analysis (**Figure 1**) for all phenotypic data was performed in R version 4.1.2 (https://www.r-project.org/).

### 2.2 Genotypic data

The genomic DNA of each sample from 156 tested accessions was isolated from a young leaf by the method of CTAB (Han et al., 2015). NanoDrop™ One (Thermo Fisher, Inc. USA) tests the quality and concentration of DNA, and the DNA OD260/OD280 was considered qualified within the range of 1.8–2.0. Each DNA was partially sequenced using the specific locus amplification fragment sequencing (SLAF-seq) method. Mse I and Hae III enzymes were used to digest soybean genomic DNA into more than 50,000 sequencing tags. The Short Oligonucleotide Alignment Program 2 (SOAP2) was used to map raw paired-end reads onto the reference genome (Glycine_max_Williams_82 8 × Release v1.01) (Li et al., 2009). According to the principle that the MAF ≥ 0.05 and missing data ≤ 1 0%, 23,150 high-quality SNPs were obtained, which were evenly distributed on 20 chromosomes of soybean.

### 2.3 Population structure evaluation and linkage disequilibrium analysis

The population structure of 156 soybean resources was analyzed using a principal component analysis (PCA) approach in the GAPIT software package (Lipka et al., 2012). The linkage disequilibrium (LD) between the pairs of SNPs was estimated using squared allele frequency correlations (r^2^) in TASSEL version 3.0 (Bradbury et al., 2007). Only SNPs with a MAF ≥ 0.05 and missing data ≤ 10% were used to estimate (\LD. In contrast to the GWAS, missing SNP genotypes were not imputed with the major allele prior to LD analysis. Parameters in the program included MAF (≥0.05) and the integrity of each SNP (≥80%).

### 2.4 Genome-wide association analysis of resistance to soybean cyst nematode HG type 0 and HG Type 1.2.3.5.7 in soybean

The GWAS of 156 soybean germplasm and 23,150 SNPs was carried out by the 3VmrMLM model. The 3VmrMLM software was downloaded from GitHub (https://github.com/YuanmingZhang65/IIIVmrMLM). The significant threshold value for the association between SNP and traits were determined by −log10 ^(P)^ ≥ 4, which is equivalent to P ≤ 0.0001. We used the single-environment and multiple-environment methods to identify QTLs and QEIs. The significant threshold value was determined by the LOD score ≥ 3. Visualization used scripts from the R package ‘qqman’ (https://cran.r-project.org/package=qqman) and ‘CMplot’ (https://github.com/YinLiLin/R-CMplot).

### 2.5 Identification of candidate genes

The candidate genes were located in the 200 kb genomic region (100 kb upstream and 100 kb downstream) of each significant or suggested QTL and the identification and annotation of candidate genes were done using the soybean reference genome (Wm82.a2.v1, http://www.soybase.org) (Cheng et al., 2017). We used online software (https://www.omicshare.com/tools/Home/Soft/gogseasenior) for gene ontology (GO) enrichment analysis of candidate genes, and we used another online software (https://www.omicshare.com/tools/Home/Soft/pathwaygseasenior) for the Kyoto Encyclopedia of Genes and Genomes (KEGG) analysis of candidate genes.

The transcriptome data from the roots of SCN-resistant cultivars ‘Dongnong L-10’ and SCN-susceptible cultivars ‘Heinong 37’ were used to further screen candidate genes, and the transcriptome data of two cultivated were from Jiang et al. (2020). The differentially expressed genes (DEGs) between the two groups were screened by P < 0.05 and then intersected with genes around significant/suggested QTLs or QEIs to obtain DEGs significantly associated with the target traits. These crossed genes are identified as candidate genes.

### 2.6 Identification of candidate genes by qRT-PCR

‘Dongnong L-10’ and ‘Heinong 37’ were used as resistant and susceptible cultivars. The seeds of soybean were germinated in vermiculite and peat soil (1:1). Plants were grown under long-day conditions (16 h light/8 h dark) at 25°C ± 1°C and relative humidity: 60%–70% for routine maintenance. HG Type 0 and HG Type 1.2.3.5.7 were obtained from the Soybean Research Institute of Northeast Agricultural University and were isolated and purified for many generations. A randomized complete block design (RCBD) was utilized, with three replicates and 10 seedlings per replicate. Each seedling was inoculated with approximately 2,000 second-stage juvenile nematodes (J2s). Accordingly, mock inoculation with distilled water was also conducted for each line as a control. All treatments and controls were watered daily to maintain soil moisture and promote uniform infection throughout the root system. Both SCN-inoculated and mock-inoculated root samples were harvested at 10-day postinoculation (dpi). The RNA extraction methods were described previously (Jiang et al., 2021). The extracted complete RNA was reverse- transcribed into cDNA. DEGs were selected and verified by qRT-PCR based on SYBR Green PCR Master Mix (TIANGEN BIOTECH, Beijing, China) and the 7500 Fast PCR detection system. The relative mRNA level of each candidate gene was evaluated against soybean *GmACTIN* (GenBank Accession Number AF049106) as a reference gene. Three technical replicates were performed per gene, and the relative levels of transcript abundance were calculated using the 2^−ΔΔCT^ method (Jain et al., 2016). The sequences of the primer pairs were used to amplify the candidate genes (**Table S6**).

### 2.7 Haplotype analysis

We selected 20 soybean cultivars (10 extreme SCN-resistant cultivars and SCN-susceptible cultivars) for genome resequencing to detect the SNP differences within and upstream (2 kb) of the candidate genes and analyzed the association of these SNPs with soybean FI after SCN stress. When log-^10(P)^ ≥ 2, it was determined that the SNPs related to the target shape reached a significant level.

### 2.8 Statistical analysis

A descriptive statistical analysis of phenotypic data including mean, minimum, maximum, the coefficient of variation (CV), heritability, skewness, and kurtosis was performed using IBM SPSS statistics 25.0 (SPSS, Chicago, IL, USA). One-way ANOVA with Dunnett’s multiple comparison test and unpaired two-tailed t-test were performed using GraphPad Prism 9.4.1.

## 3 Result

### 3.1 Phenotypes of soybean materials under soybean cyst nematode HG Type 0 and HG Type 1.2.3.5.7 stressed

The FI of 156 soybean materials under SCN HG Type 0 and HG Type 1.2.3.5.7 stress was analyzed by the Pearson correlation coefficient (**Figure 1**). FI varies widely, from 0% to 308.76%, and its distribution is close to normal distribution. Under the same physiological race stress, the FI in different environments showed a positive correlation, among which the FI of Jilin and Liaoning was the most correlated (PCC = 0.932) under SCN HG Type 0 stress. In the same environment, only the FI of soybean materials grown in Jilin was positively correlated under the stress of two different SCN physiological races, and the others were not correlated. These results showed that the resistance of soybean materials to SCN varied greatly among different physiological races and was less affected by environmental factors. Therefore, the average FI values in this study were used as phenotypic data for GWAS.

### 3.2 Analysis of genotypic data of soybean germplasm resources

**Figure 2:**
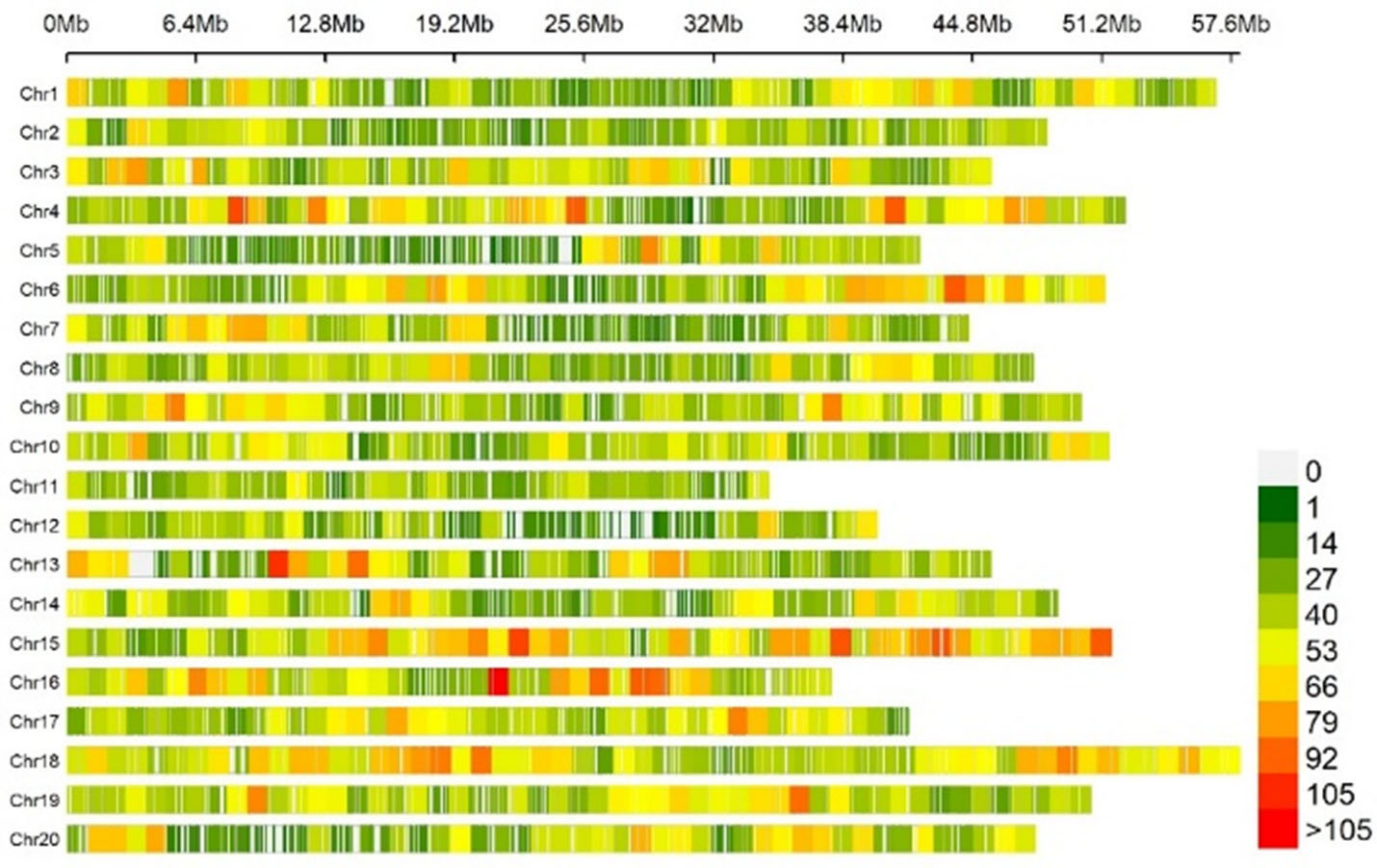
The density distribution of single-nucleotide polymorphisms (SNPs).

A total of 156 soybean resources were genotyped by specific-locus amplified fragment sequencing (SLAF-seq). A total of 23,150 high-quality SNPs were obtained according to the principle that the minor genotype frequency (MAF) was more than 0.05 and the missing rate was less than 0.1. These SNPs are distributed on 20 chromosomes of soybean (**Figure 2**); the number of SNP markers on each chromosome is uneven, and the rate of chromosome variation is high. Among them, the number of markers on chromosome 11 was the lowest, only 783 SNP markers; chromosome 18 was the most, reaching 18,951.

**Figure 3:**
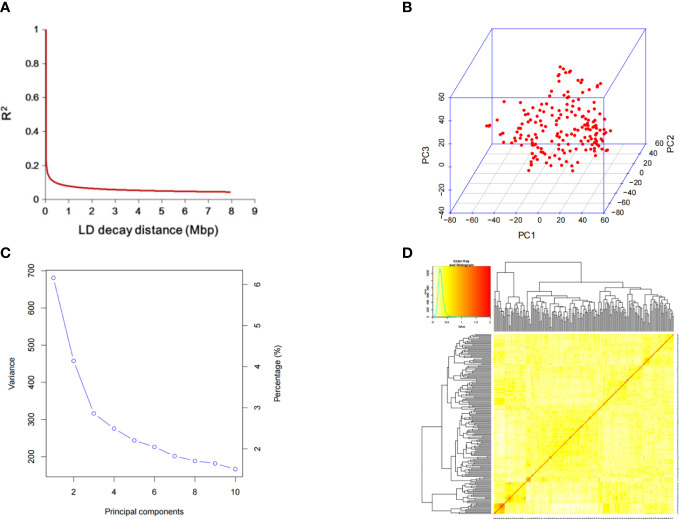
SNP distribution and mapping genetic data of populations. **(A)** linkage disequilibrium (LD) decay of the genome-wide association study (GWAS) population. **(B)** Population structure of soybean germplasm collection reflected by principal components. **(C)** The first three principal components of the 23,150 SNPs used in the GWAS. **(D)** A heatmap of the kinship matrix of the 156 soybean accessions.

The decline distance of average LD was 212 kbp, indicating that the genetic diversity of these soybean materials was high(**Figure 3A**). Principal component and kinship analyses were performed using the entire set of SNPs to capture the overall population stratification of the association panel. The first three PCs dominated the population structure on the association map in the 10 PC analyses (**Figures 3B, C**). The heat map of soybean genetic relationship matrix and the genetic relationship calculated by 23,150 SNPs of 156 soybean materials showed that the level of genetic relationship between each individual was low (**Figure 3D**).

### 3.3 Quantitative trait locus associated with soybean cyst nematode HG Type 0 and HG Type 1.2.3.5.7 by genome-wide association study

**Figure 4:**
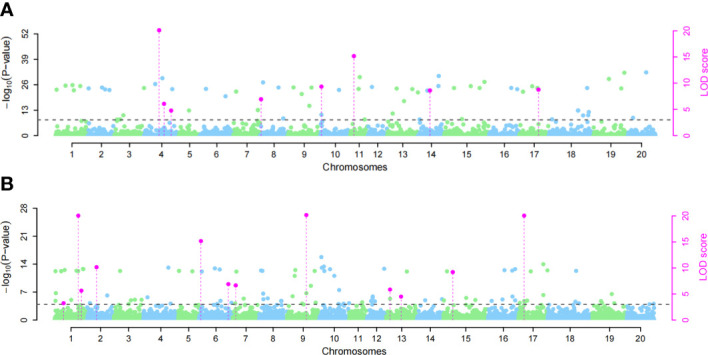
Manhattan plots of the GWAS for soybean susceptibility to HG Type 0 and HG Type 1.2.3.5.7 in Harbin. **(A)** GWAS for HG Type 0, **(B)** GWAS for HG Type 1.2.3.5.7. Significant quantitative trait locus (QTL) in phenotypic GWAS was set at −log10^(P)^ ≥ 4 and LOD ≥ 3 for the 3VmrMLM as the significant QTL, and all the critical values were marked by horizontal lines. The Y-axis on the left side reports –log10^(P-values)^ of SNP, while the Y-axis on the right side reports LOD scores, and LOD scores are shown in points with straight lines.

We used the single-environment detection model and multienvironment detection model in 3VmrMLM to GWAS the phenotypic values of two SCN physiological races under stress. The QTL associated with SCN resistance was detected in three environments, and we used different significant thresholds (By −log10^(P)^ ≥ 4 or LOD score ≥ 3) for analysis (**Figures 4** and **S1–S4**). Through the single-environment detection model, 53 QTLs were detected to be associated with HG Type 0 and HG Type 1.2.3.5.7 resistance (**Table S2**). Among them, 28 QTLs were related to HG Type 0, 27 QTLs were related to HG Type 1.2.3.5.7, 36 QTLs were detected by the multienvironment detection model (**Table S3**), of which 17 QTLs were related to HG Type 0, and 19 QTLs were related to HG Type 1.2.3.5.7. We selected the QTL located in at least two models or two environments as reliable QTL. A total of 18 QTLs were detected by at least two models together (**Table 1**). The value of −LOG10^(p)^ was 4.18–33.79, distributed on 12 chromosomes. Among these, eight QTLs (rs27634423, rs46410067, rs32462952, rs22236673, rs23148030, rs11720920, rs33704130, and rs47402736) have been reported to be related to SCN in previous studies, which also confirms the accuracy of our studies. We regard the remaining 10 QTLs (rs35111254, rs17071358, rs456656, rs50885278, rs5726664, rs15274949, rs24026375, rs15049524, rs18628696, and rs22586702) as novel QTLs, among which there were 5 QTLs related to HG Type 0 and 5 QTLs related to HG Type 1.2.3.5.7. Two QTLs, rs33704130 on G6 and rs456656 on Ghr 3, were significantly related to the resistance of the two SCN physiological races. In our previous study, rs33704130 on Ghr16 was identified to be associated with resistance to SCN HG Type 0 and HG Type 1.2.3.5.7 (Han et al., 2015), while rs456656 on Ghr 3 was identified for the first time to be associated with resistance to SCN HG Type 0 and HG Type 1.2.3.5.7, which explained 8%–19.55% of the phenotypic variation.

**Table 1:** Peak single-nucleotide polymorphisms (SNPs) associated with resistance to the HG Type 0 and HG Type 1.2.3.5.7 identified by the genome-wide association study (GWAS).

SNP	Chr.	Position (bp)	Allele	HG type	Environment	LOG10(P)	r^2^ (%)	significance	References
rs27634423	1	27634423	T/A	race 4	Jilin	5.374715419	3.4682	SUG	Yue et al., 2000
					Liaoning	12.78240103	8.4174	SIG	
					Multiple-env	8.573857758	1.7176	SIG	
rs35111254	1	35111254	G/T	race 3	Jilin	7.829287052	5.5926	SIG	
					Liaoning	9.782185141	6.757	SIG	
					Multiple-env	10.47147187	3.0638	SIG	
rs17071358	2	17071358	C/A	race 4	Liaoning	6.88293967	3.8474	SIG	
					Multiple-env	9.406455936	1.6479	SIG	
rs456656	3	456656	T/C	race 4	Jilin	20.47366724	19.5574	SIG	
				race 4	Liaoning	13.90300662	9.3462	SIG	
				race 3	Harbin	16.35	15.6	SIG	
				race 4	Multiple-env	33.79189902	8.0233	SIG	
rs46410067	4	46410067	A/G	race 3	Jilin	19.62125827	9.0298	SIG	Valdes-Lopez et al., 2011a
					Liaoning	20.09048066	7.7372	SIG	
					Multiple-env	17.87182633	2.5986	SIG	
rs50885278	4	50885278	A/G	race 4	Jilin	7.798927031	5.8871	SIG	
					Liaoning	6.162662476	3.6043	SIG	
					Multiple-env	4.18543368	0.827	SIG	
rs32462952	5	32462952	G/A	race 3	Jilin	7.415523124	5.2512	SIG	Yue et al., 2001
					Liaoning	9.99797881	5.8936	SIG	
					Multiple-env	8.114855718	1.3517	SIG	
rs5726664	6	5726664	G/T	race 3	Jilin	9.470874794	7.7936	SIG	
					Liaoning	11.31226562	7.3874	SIG	
					Multiple-env	14.75865408	4.3645	SIG	
rs22236673	9	22236673	G/A	race 3	Liaoning	7.869201836	4.5058	SIG	Wu et al., 2009
					Multiple-env	4.825021146	1.393	SIG	
rs15274949	13	15274949	A/G	race 3	Liaoning	11.97119859	8.0165	SIG	
					Multiple-env	4.636874623	0.9425	SIG	
rs23148030	13	23148030	G/T	race 4	Liaoning	6.534465041	3.6106	SIG	Valdes-Lopez et al., 2011a
					Multiple-env	15.6361376	3.4247	SIG	
rs24026375	13	24026375	G/T	race 4	Jilin	8.373451852	6.4348	SIG	
					Liaoning	7.397039816	4.6667	SIG	
					Multiple-env	27.20264692	2.9766	SIG	
rs15049524	14	15049524	C/T	race 4	Jilin	12.22976965	10.3712	SIG	
					Liaoning	12.57969391	8.441	SIG	
					Multiple-env	7.650568005	1.5644	SIG	
rs18628696	14	18628696	C/A	race 3	Jilin	21.19107064	3.0741	SIG	
					Liaoning	28.52065381	4.0512	SIG	
rs22586702	14	22586702	G/T	race 4	Liaoning	6.040995322	3.7296	SIG	
					Multiple-env	18.75686438	4.1102	SIG	
rs11720920	15	11720920	C/A	race 4	Jilin	22.03505536	3.9921	SIG	Kabelka et al., 2005
					Liaoning	26.56943573	4.1231	SIG	
rs33704130	16	33704130	A/C	race 3	Harbin	8.41	7.65	SIG	Han et al., 2015
				race 4	Harbin	6.7	7.74	SIG	
rs47402736	18	47402736	T/A	race 3	Jilin	13.22463402	11.759	SIG	Winter et al., 2007
					Liaoning	9.351008329	6.451	SIG	
					Multiple-env	15.075767	4.5547	SIG	

SIG represents significant QTLs, and SUG represents suggested QTLs. HG Type 0 (race 3) and HGType 1.2.3.5.7(race 4)

### 3.4 Detection of quantitative trait locus-by-environmental interactions related to soybean cyst nematode resistance by 3VmrMLM

In multienvironment analysis, the resistance to SCN in three sites (Harbin, Liaoning, and Jilin) was analyzed by using 3VmrMLM QEIs detection model to identify QEIs. A total of nine QEIs associated with SCN resistance were detected (**Figure S4** and **Table S4**), Among these QEIs, distributed on eight chromosomes, six QEIs have been reported to be associated with SCN resistance in previous studies. Among the remaining three QEIs, rs15049524 on chromosome 14 was related to HG Type 1.2.3.5.7, and rs14265606 on chromosome 13 was related to HG Type 0. The rs456656 on chromosome three was associated with two physiological races of SCN, which was the same as our previous mapping results in the single-environment model; thus, the rs456656 on Ghr 3 might be an important QTL for resistance to multiple physiological races of SCN.

### 3.5 Gene-based association analysis of candidate genes

**Figure 5:**
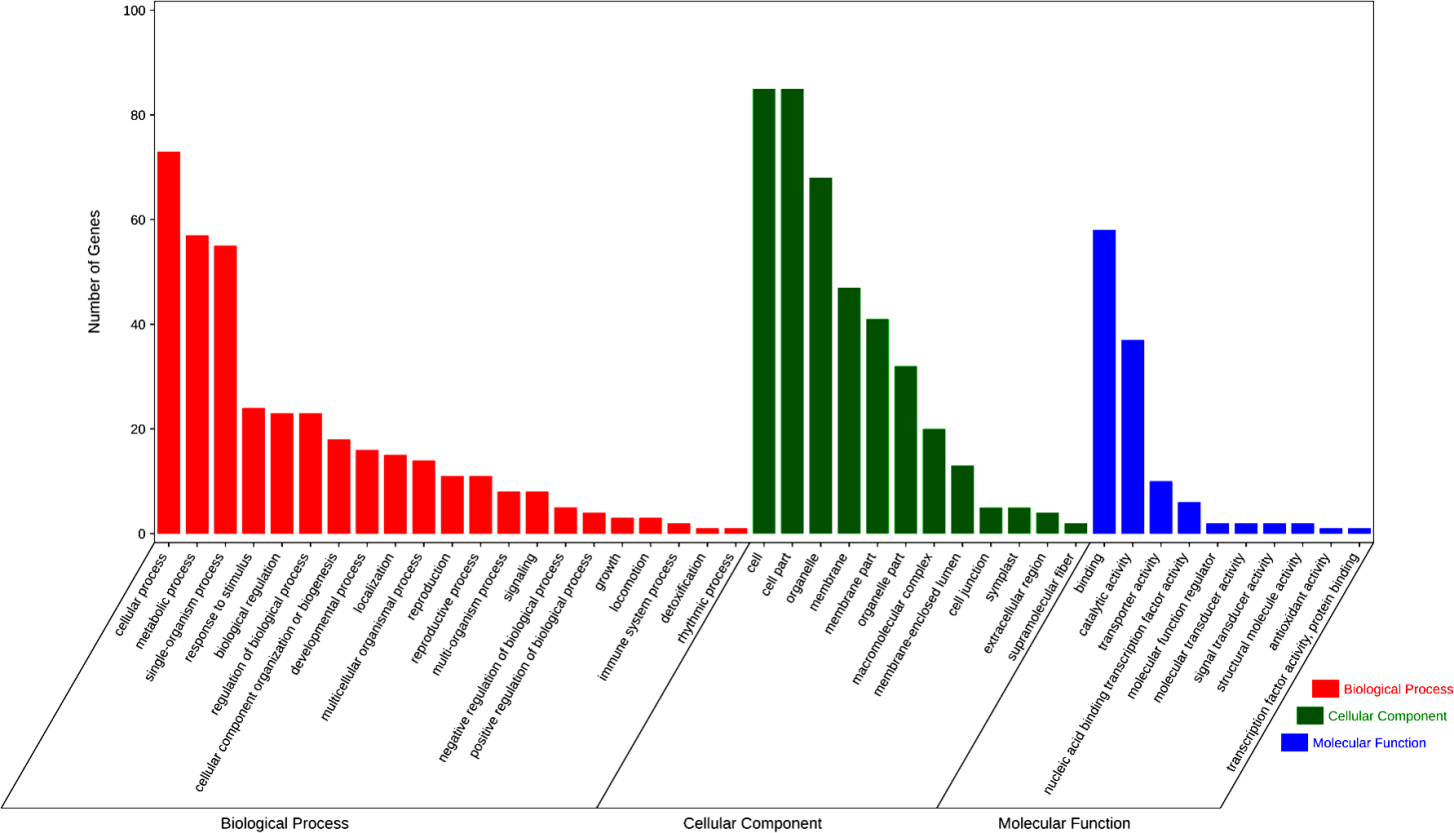
Gene ontology term enrichment analysis of candidate genes.

As two of the three new QEIs coincide with the QTLs located by single-environment and multiple-environment models, we finally got 10 novel QTLs and one QEI, which have not been reported in previous studies. We further evaluated the identified QTL-related candidate genes. Since the average LD decay distance of soybean genes was about 200 kb, we defined the genes in the upstream and downstream 100 kb regions of each QTL as candidate genes for disease resistance (**Table S5**). A total of 144 soybean genes were found in these QTL-flanking regions. There were 43 of these genes that were not annotated, while the other 101 genes were classified by GO annotation (**Figure 5**), which were divided into 44 subgroups in terms of molecular function, cell composition, and biological processes. Among them, cellular process and metabolic process genes were the most abundant, while binding, cell, and cell part had the most genes in cell composition and biological processes.

In the KEGG analysis of candidate genes, these candidate genes are related to 32 metabolic pathways (**Figure S5**). Some of these genes were involved in tyrosine metabolism, ABC transporters, the MAPK signaling pathway—plant, SNARE interactions in vesicular transport, plant–pathogen interaction, and the plant hormone signal transduction pathway, and these metabolic pathways have been proven to be related to plant disease resistance or SCN resistance (Bayless et al., 2018; Jiang et al., 2020; Jiang et al., 2021). In these gene families, six genes containing the MATE efflux family protein domain were found on both sides of rs456656 of Ghr 3, which may be related to the resistance of HG Type 0 and HG Type 1.2.3.5.7.

### 3.6 Screening of candidate genes using RNA-seq data of soybean cyst nematode–resistant and soybean cyst nematode–susceptible cultivars

**Figure 6:**
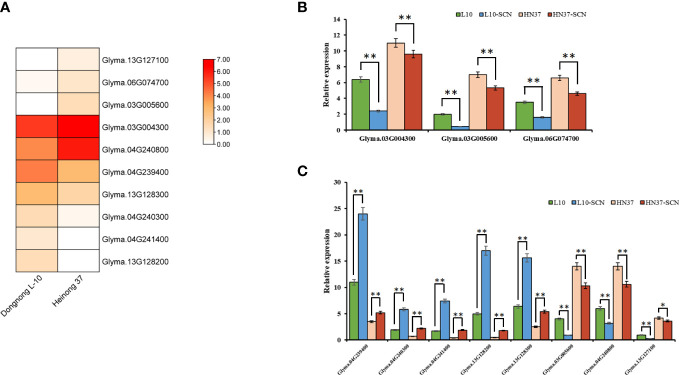
The expression of 10 key candidate genes. **(A)** Expression profile of 10 key candidate genes in the RNA-data of SCN-resistant cultivars ‘Dongnong L-10’ and SCN-susceptible cultivars ‘Heinong 37’. **(B)** Expression differences of three key genes in SCN-resistant and SCN-susceptible cultivars before and after HG Type 0 stress. **(C)** Expression differences of eight key genes in SCN-resistant and SCN-susceptible cultivars before and after HG Type 1.2.3.5.7 stress (Student’s *t*-test: **p < 0.01, *p < 0.05).

In order to further screen candidate genes, the differential analysis of the RNA-seq data of the SCN-resistant cultivars ‘Dongnong L-10’ and the SCN-susceptible cultivars ‘Heinong37’ showed that 5,919 DEGs were obtained. There were 10 candidate genes that were obtained by being intersected with DEGs and candidate genes (**Figure 6A**). These genes were located on Chr 3 (rs456656), Chr 4 (rs50885278), Chr 6 (rs5726664), and Chr 13 (rs24026375), and the expression of gene-encoding dihydrofolate reductase-like (Glyma.03G004300), MATE efflux family protein (Glyma.03G005600), alcohol dehydrogenase 1 (Glyma.04G240800), and leucine-rich repeat receptor-like protein kinase (Glyma.13G127100) in SCN-resistant cultivars was lower than that in SCN-susceptible cultivars. The expression of gene-encoding protein MKS1-like (Glyma.04G239400), transmembrane protein (Glyma.04G240300), bidirectional sugar transporter SWEET1-like (Glyma.04G241400), receptor-like serine/threonine-protein kinase (Glyma.13G128200), and acyl-activating enzyme (Glyma.13G128300) in SCN-resistant cultivars was higher than that in SCN-susceptible cultivars. In order to verify the accuracy of these 10 candidate genes, the expression of 10 genes were measured in SCN-resistant and SCN-susceptible cultivars before and after HG Type 0 and HG Type 1.2.3.5.7 stress, and the results showed that the expression of 10 genes were up- and downregulated under SCN stress in SCN-resistant and SCN-susceptible cultivars. The change trend of expression was the same as that in RNA-seq date, which reflects the accuracy of transcriptome data. Furthermore, the expression of Glyma.06G074700, Glyma.03G004300 and Glyma.03G005600 genes after HG Type 0 stress in resistant cultivars increased and decreased more than that in susceptible cultivars (**Figure 6B**). Similarly, the expression of Glyma.03G005600, Glyma.04G239400, Glyma.04G240300, Glyma.04G240800, Glyma.04G241400, Glyma.13G127100, Glyma.13G128200, and Glyma.13G128300 after HG Type 1.2.3.5.7 stress in SCN-resistant cultivars increased and decreased more than that in SCN-susceptible cultivars (**Figure 6C**). Interestingly, the expression of Glyma.03G005600 was downregulated after HG Type 0 and HG Type 1.2.3.5.7 stress; thus, we speculated that the MATE efflux family protein was involved in the resistance to HG Type 0 and HG Type 1.2.3.5.7.

### 3.7 Haplotype and phenotypic difference analysis of candidate genes

**Figure 7:**
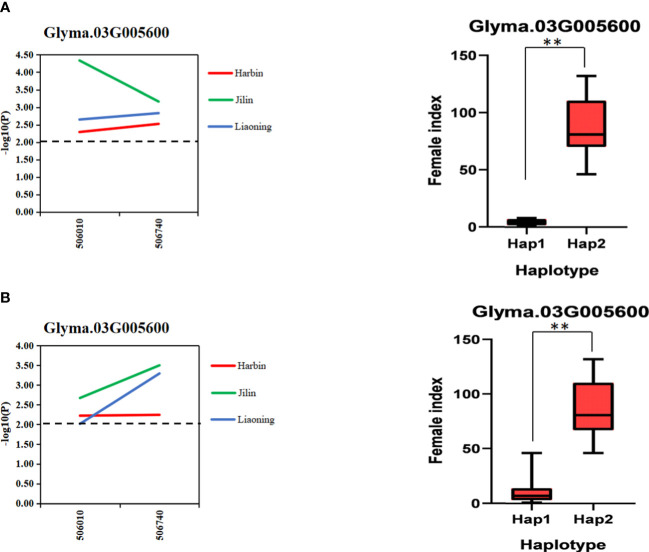
Gene-based association analysis and haplotype analysis. **(A)**. Association analysis and haplotype analysis of candidate genes related to HG Type 0 resistance **(B)**. Association analysis and haplotype analysis of candidate genes related to HG Type 1.2.3.5.7 resistance. Horizontal line indicates that the threshold is set to 2.0; the ** mean significance at P < 0.05 and P < 0.01.

In order to further verify the association between candidate genes and target traits, the 612 SNPs of 10 candidate genes were obtained through the association analysis of candidate genes for the resequencing data of 20 soybean lines (10 lines with higher/lower resistance levels of HG Type 0 and HG Type 1.2.3.5.7). Among them, 20 SNPs of 10 candidate genes reached the threshold of −log10^(P)^ ≥ 2 (**Table 2**). The haplotype analysis of candidate genes and its SNPs of 2 kb upstream showed that each of the 10 genes has at least two haplotypes, and the FI values of these different haplotypes has significant or extremely significant differences (**Figures 7**, **S6**, and **S7**). Among them, Glyma.03G005600 has two significant haplotypes in HG Type 0 and HG Type 1.2.3.5.7. In other words, Glyma.03G005600 has a beneficial haplotype with resistance to two physiological races; thus, these haplotypes are beneficial. We speculate that SNPs lead to the DEGs of candidate genes based on these results. The discovery of these candidate genes is of great significance for improving the efficiency of marker-assisted breeding and exploring the molecular mechanism of disease resistance.

**Table 2:** Haplotype analysis of candidate genes.

Gene ID	Chromosome	Physical position (bp)	HG type	Location	Region	Alleles	−log10(P)	Functional annotation
Glyma.03G004300	3	378908	Race 3	Harbin	Upstream	G/A	2.32	Dihydrofolate reductase-like
				Jilin			1.08	
				Liaoning			0.50	
		378978	Race 3	Harbin	Upstream	T/G	2.24	
				Jilin			1.44	
				Liaoning			1.29	
Glyma.03G005600		506010	Race 3	Harbin	Intronic	C/T	2.29	MATE efflux family protein
				Jilin			4.34	
				Liaoning			2.65	
			Race 4	Harbin	Intronic	C/T	2.22	
				Jilin			2.67	
				Liaoning			2.01	
		506740	Race 3	Harbin	Non-synonymous	C/G	2.52	
				Jilin			3.16	
				Liaoning			2.83	
			Race 4	Harbin	Non-synonymous	C/G	2.24	
				Jilin			3.50	
				Liaoning			3.29	
Glyma.04G239400	4	50794074	Race 4	Harbin	Upstream	C/T	1.54	Protein MKS1-like
				Jilin			2.65	
				Liaoning			2.34	
		50794174	Race 4	Harbin	Upstream	C/A	2.36	
				Jilin			3.20	
				Liaoning			1.70	
Glyma.04G240300	4	50871855	Race 4	Harbin	Upstream	T/G	3.95	Transmembrane protein
				Jilin			2.25	
				Liaoning			1.24	
		50873030	Race 4	Harbin	Upstream	T/A	1.57	
				Jilin			3.95	
				Liaoning			2.01	
Glyma.04G240800	4	50912427	Race 4	Harbin	Upstream	G/A	3.95256936	Alcohol dehydrogenase
				Jilin			2.22	
				Liaoning			2.01	
		50917739	Race 4	Harbin	Non-synonymous	A/C	1.80	
				Jilin			3.20	
				Liaoning			2.20	
Glyma.04G241400	4	50940339	Race 4	Harbin	Upstream	G/A	2.40	Bidirectional sugar transporter SWEET1-like
				Jilin			2.12	
				Liaoning			2.01	
		50940763	Race 4	Harbin	Upstream		2.30	
				Jilin			2.22	
				Liaoning			2.80	
Glyma.06G074700	6	5761034	Race 3	Harbin	Intronic	C/A	2.79	Hypothetical protein
				Jilin			4.12	
				Liaoning			4.01	
		5762360	Race 3	Harbin	Non-synonymous	A/C	2.04	
				Jilin			3.10	
				Liaoning			2.00	
Glyma.13G127100	13	24019905	Race 4	Harbin	Upstream	C/T	2.67	Leucine-rich repeat receptor-like protein kinase
				Jilin			1.20	
				Liaoning			2.08	
		24019876	Race 4	Harbin	Upstream	C/G	2.22	
				Jilin			1.66	
				Liaoning			1.24	
Glyma.13G128200	13	24093346	Race 4	Harbin	Upstream	C/T	2.46	Receptor-like serine/threonine–protein kinase
				Jilin			1.19	
				Liaoning			2.78	
		24093233	Race 4	Harbin	Upstream	A/T	2.12	
				Jilin			1.90	
				Liaoning			2.78	
Glyma.13G128300	13	24121928	Race 4	Harbin	Upstream	A/G	2.34	Acyl-activating enzyme
				Jilin			3.54	
				Liaoning			1.80	
		24121826	Race 4	Harbin	Upstream	A/C	2.45	
				Jilin			2.20	
				Liaoning			2.05	

HG Type 0 (race 3) and HGType 1.2.3.5.7 (race 4).

## 4 Discussion

SCN is one of the diseases causing a serious loss of soybean yield (Cook et al., 2012). The resistance mechanism of soybean to SCN is complex, and classical quantitative genetics has increasingly highlighted its limitations in SCN resistance breeding (Yue et al., 2001). The total number of SCN-resistant varieties in soybean production was relatively small, and almost all of them are directly or indirectly derived from a few small black bean varieties. Most of these SCN-resistant cultivars were only resistant to a single physiological race, in another word, a single race-resistant variety (Han et al., 2015). The long-term planting of single resistant varieties has led to the transfer of SCN resistance, from a single physiological race to a mixture of multiple physiological races (Han et al., 2015). Multirace-resistant varieties (horizontally resistant or non-specific resistant varieties) have no specific resistance or specific response to a single (or specific) physiological race; thus, there is no selection pressure on a single physiological race, which will not cause rapid variations of the SCN physiological race, and its ‘resistance’ is not easy to lose (Mitchum, 2016). Therefore, it was very necessary to tap multiphysiological race resistance resources. In this study, 156 soybean germplasm resources collected in China were identified. Among these materials, 10 materials showed high resistance to HG Type 0 and HG Type 1.2.3.5.7, and most of them were local cultivars with specific excellent agronomic characters. Therefore, these resistance sources can provide useful resources for SCN resistance breeding in the future.

The GWAS based on natural populations was an alternative method of linkage analysis and was widely used in the analysis of important crop traits (Rakotoson et al., 2021; Yuan et al., 2021). Compared with linkage analysis, the range of phenotypic variation in single analysis was increased by GWAS, which was due to the high natural variation caused by the accumulation of historical reorganization events in the natural population (Liu et al., 2017). The development of genome sequencing and SNP genotyping technology has promoted the applicability of the GWAS in soybean research; thus, it was particularly important to select an appropriate model for effective and accurate mapping according to research needs (Kim et al., 2021). The mixed linear model (MLM) and the general linear model (GLM) was a classic GWAS model. However, due to its strict threshold, many small effect QTLs are lost, which leads to the neglect of the overall response of multiple loci (Zhang et al., 2005). Therefore, geneticists have developed a multilocus genetic structure model that can reduce the bias related to the estimation effect by controlling the population structures and polygenic background and improve the ability to detect QTLs (Zhang et al., 2005; Zhang et al., 2010). In this study, we located 18 reliable QTLs by using the 3VmrMLM model to detect HG Type 0 and HG Type 1.2.3.5.7 resistance of 156 soybean germplasm resources by QTLs. Eight of these QTLs (rs27634423, rs46410067, rs32462952, rs22236673, rs23148030, rs11720920, rs33704130, and rs47402736) overlap with or near known QTLs, and the relationship between these genomic regions and SCN resistance has been reported in previous studies (Yue et al., 2001; Kabelka et al., 2005; Winter et al., 2007; Wu et al., 2009; Valdes-Lopez et al., 2011; Han et al., 2015). At the same time, we detected 10 novel QTLs that have never been published before, which reflected the accuracy of the 3VmrMLM method and the advantages compared with other models.

Many large QTL intervals can be obtained by GWAS, but it was very difficult to screen candidate genes from large QTL intervals. Although we can narrow the range by using LD blocks (the average length is 150–200 kb), there were still many genes in the reduced range; thus, the screening of candidate genes has always been a challenge (Lam et al., 2010). At present, with the rapid development of the second-generation sequencing technology, many satisfactory results have been obtained by using multiomics methods to mine candidate genes (Liu et al., 2022). In this study, we detected that there were 144 candidate genes located in 11 QTLs (upstream and downstream:100 kbp). Candidate genes were screened by using transcriptome sequencing data of SCN-resistant cultivars ‘Dongnong L-10’ and SCN-susceptible cultivars ‘Heinong 37’. A total of 10 candidate genes were obtained by intersecting 5,919 DEGs and candidate genes. qRT-PCR analysis further confirmed that these candidate genes may be involved in the response of soybean to SCN.

Among these candidate genes, the genes encoding dihydrofolate reductase–like protein (Glyma.03G004300), hypothetical protein (Glyma.06G074700), and MATE efflux family protein (Glyma.03G005600) were associated with HG Type 0 resistance, the genes encoding protein MKS1-like (Glyma.04G239400), transmembrane protein (Glyma.04G240300), alcohol dehydrogenase (Glyma.04G240800), bidirectional sugar transporter SWEET1-like (Glyma.04G241400), leucine-rich repeat receptor-like protein kinase (Glyma.13G127100), receptor-like serine/threonine-protein kinase (Glyma.13G128200), acyl-activating enzyme (Glyma.13G128300), and MATE efflux family protein (Glyma.03G005600) were associated with HG Type 1.2.3.5.7 resistance. Leucine-rich repeat receptor-like protein kinase and receptor-like serine/threonine-protein kinase are typical SCN resistance domains, which have been reported to be related to SCN resistance (Gao et al., 2009; Liu et al., 2011). Among these genes, MATE efflux family protein (Glyma.03G005600) was related to the resistance of two physiological races. It has the function of regulating plant growth and senescence, responding to disease and stress. However, there were no reports about the relationship between this gene and SCN resistance, and whether these genes have the function of SCN resistance needs further verification.

## Data availability statement

The original contributions presented in the study are publicly available. This data can be found here: https://www.ebi.ac.uk/ena/browser/view/PRJEB55008.

## Author contributions

HJ: methodology, writing—original draft, and writing—review and editing. CZ and SL: data curation. SQ, FL and HS: data curation. XZ: supervision. YH: project administration, supervision, and writing—review and editing. All authors contributed to the article and approved the submitted version.
