# Supplementary material for: Identification of QTL, QTL-by-environment interactions, and their candidate genes for resistance HG Type 0 and HG Type 1.2.3.5.7 in soybean using 3VmrMLM
Source: Front Plant Sci. 2023 Apr 21;14:1177345. doi: 10.3389/fpls.2023.1177345 (PMC10162016; doi:10.3389/fpls.2023.1177345)
Supplement: Supplementary file 1 [file DataSheet_1.docx]

**Supplementary Figures**


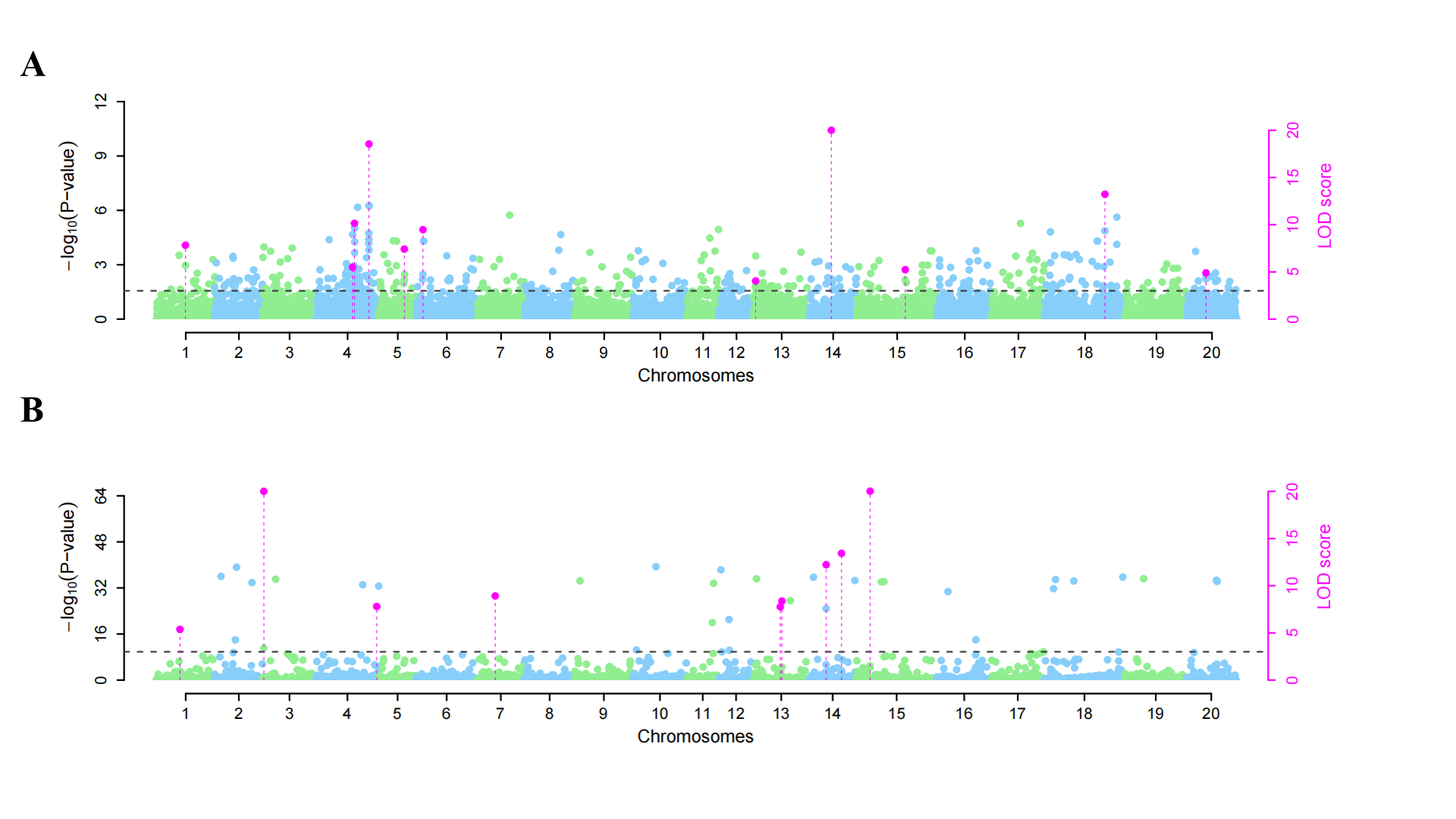


**Figure S1.** Manhattan plots of GWAS for soybean susceptibility to HG Type 0 and HG Type 1.2.3.5.7 in Jilin. (A) GWAS for HG Type 0. (B) GWAS for HG Type 1.2.3.5.7.


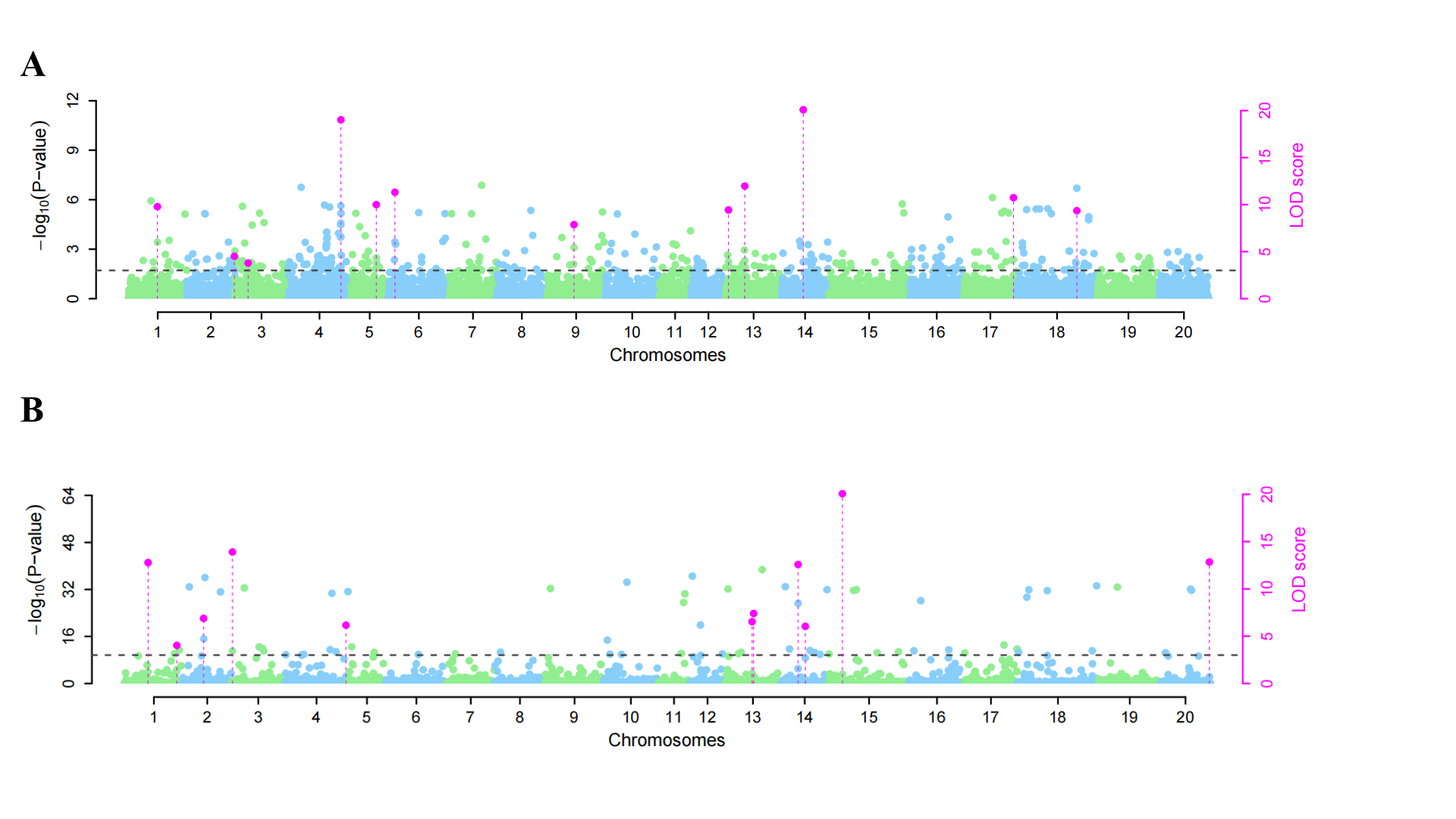


**Figure S2.** Manhattan plots of GWAS for soybean susceptibility to HG Type 0 and HG Type 1.2.3.5.7 in Liaoning. (A) GWAS for HG Type 0, (B) GWAS for HG Type 1.2.3.5.7.


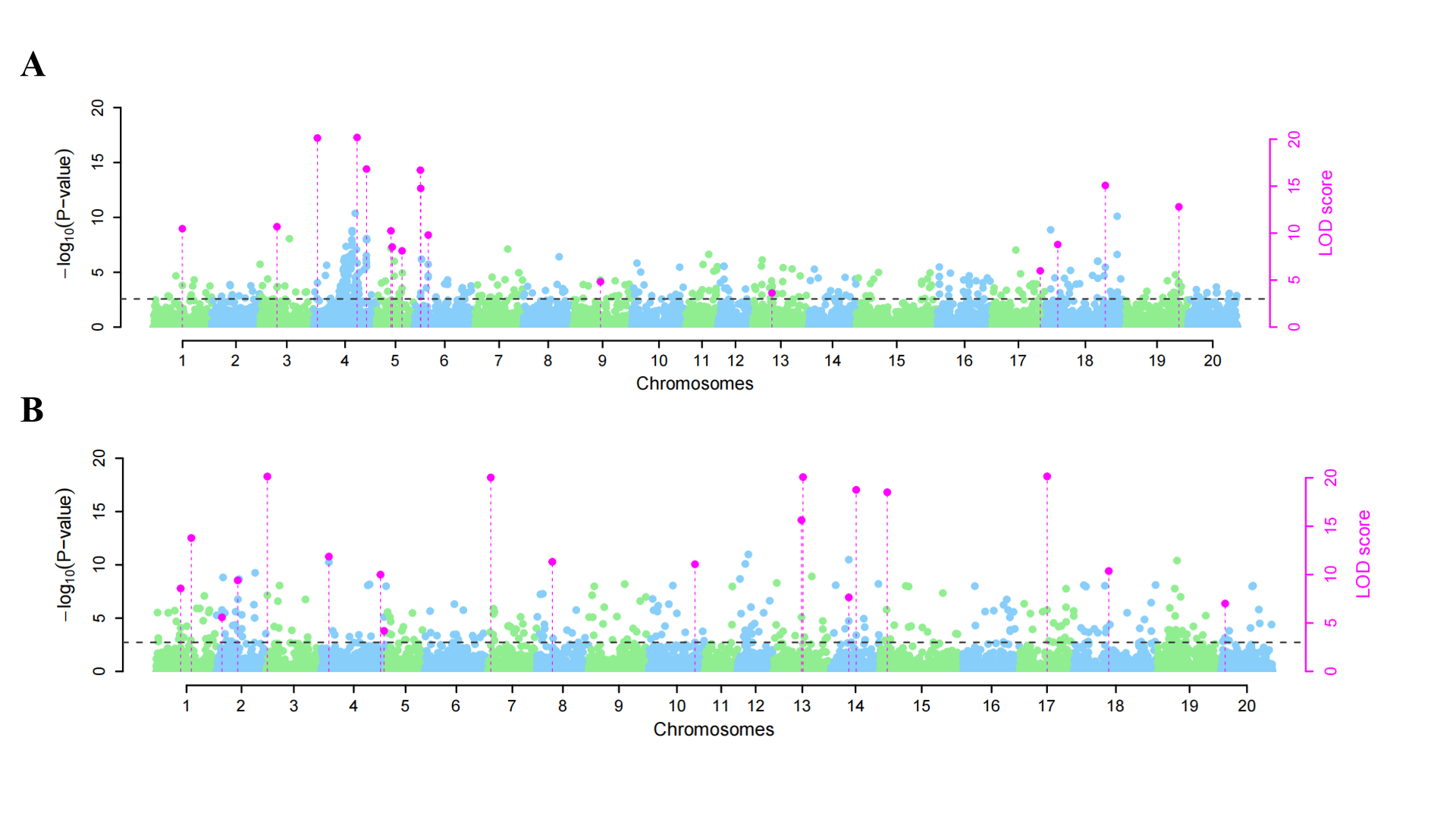


**Figure S3.** Manhattan plots of GWAS for soybean susceptibility to HG Type 0 and HG Type 1.2.3.5.7 using the 3VmrMLM model single-environment method. (A) GWAS for HG Type 0, (B) GWAS for HG Type 1.2.3.5.7.


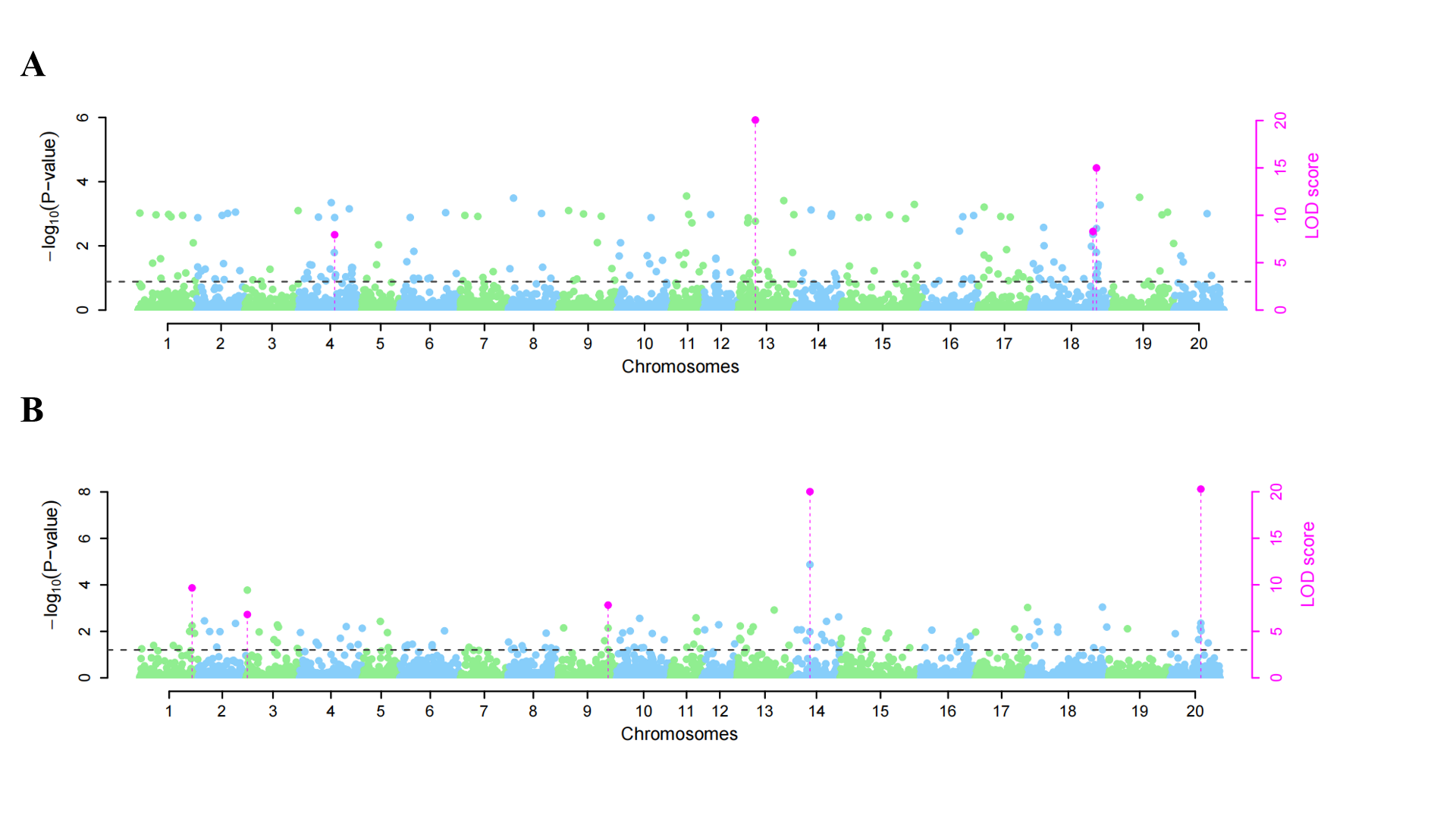


**Figure S4.** Manhattan plots of GWAS for soybean susceptibility to HG Type 0 and HG Type 1.2.3.5.7 using QEI detection of 3VmrMLM multiple-environment method. (A) GWAS for HG Type 0, (B) GWAS for HG Type 1.2.3.5.7.


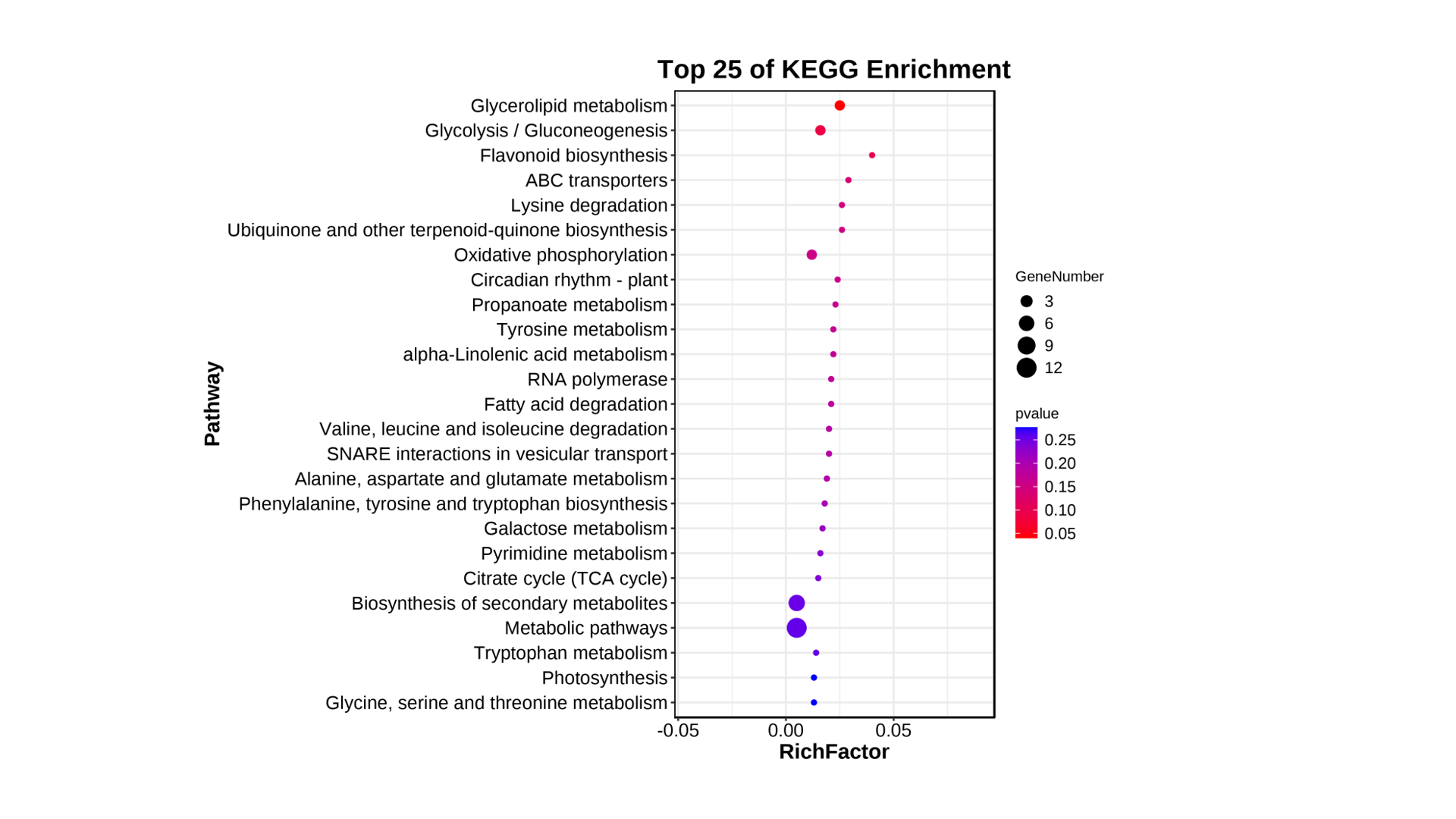


**Figure S5.** KEGG annotation of candidate genes.


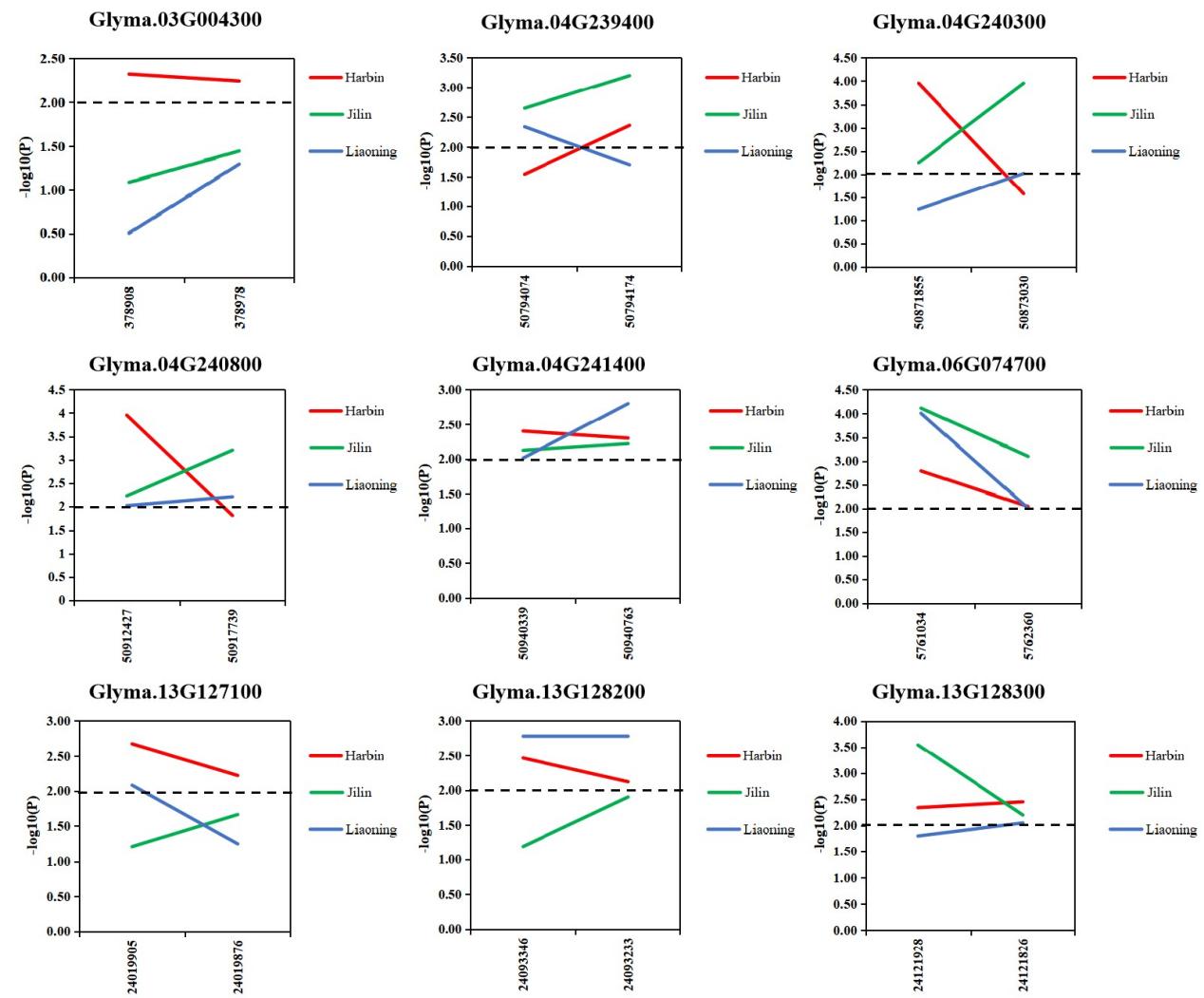


**Figure S6.** Gene-based association analysis of candidate genes. The horizontal line indicates that the threshold was set to 2.0.


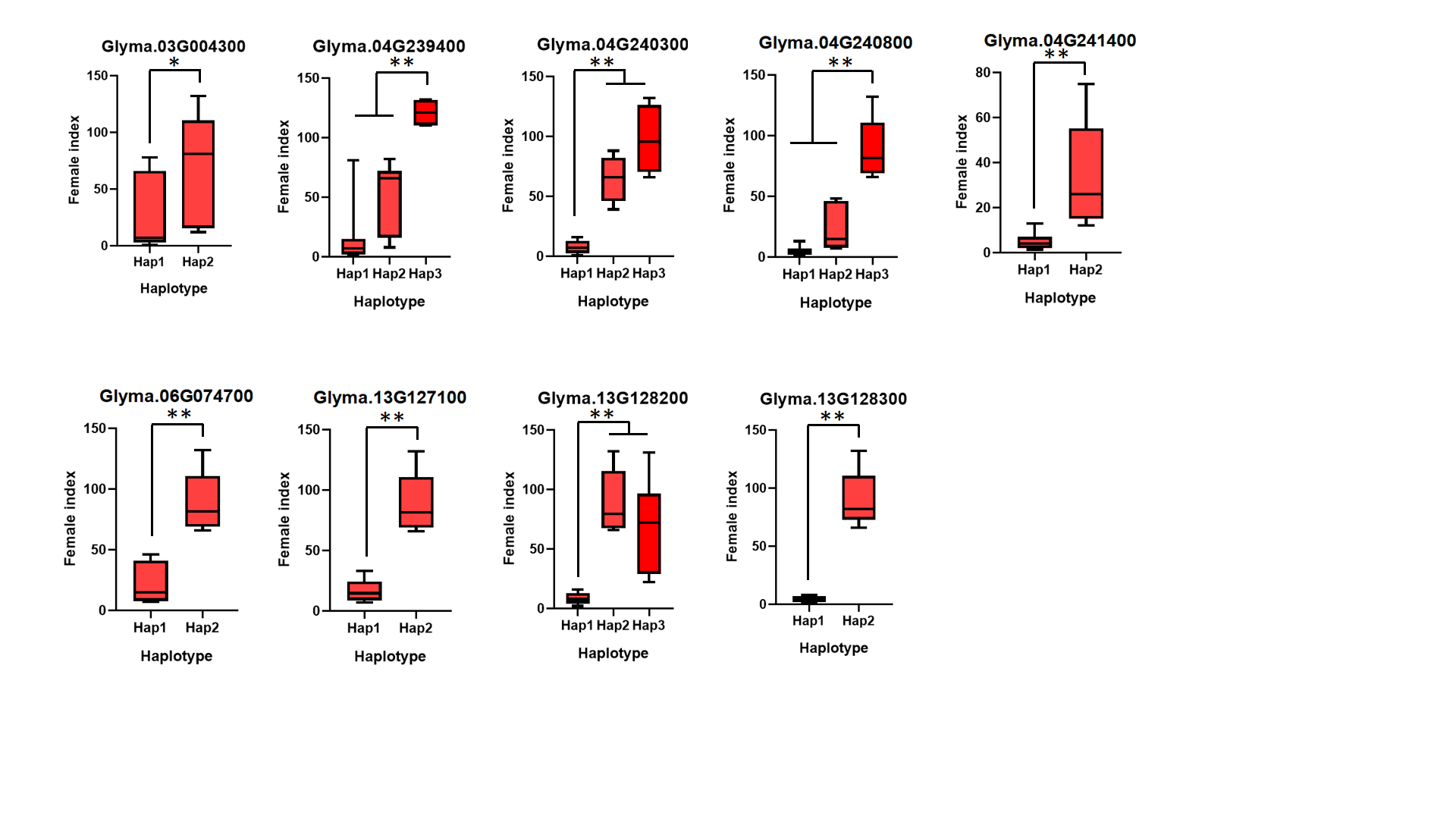


**Figure S7.** Haplotypes analysis of candidate genes that related to HG Type 0 and HG Type 1.2.3.5.7 resistance. The * and ** was significance at *p* < 0.05 and *p* < 0.01, respectively.

**Supplementary Tables**

**Table S1** The information of 156 soybean accessions.

| Name | Country | Latitude (°N) | Longitude (°W) |
| --- | --- | --- | --- |
| L089 | China | 37.34 | 100.3 |
| L076 | China | 37.34 | 84.79 |
| L015 | China | 48.29 | 128.08 |
| L036 | China | 48.29 | 128.08 |
| L090 | China | 39.19 | 116.29 |
| L095 | China | 39.19 | 116.29 |
| L154 | China | 39.19 | 116.29 |
| L169 | China | 39.19 | 116.29 |
| L101 | China | 39.19 | 116.29 |
| L176 | China | 39.19 | 116.285 |
| L177 | China | 48.29 | 128.08 |
| L178 | China | 40.145 | 116.275 |
| L174 | China | 48.29 | 128.08 |
| L096 | China | 40.15 | 116.28 |
| L171 | China | 40.145 | 116.275 |
| L172 | China | 40.145 | 116.275 |
| L085 | China | 40.15 | 116.28 |
| L086 | China | 40.15 | 116.28 |
| L163 | China | 40.15 | 116.28 |
| L098 | China | 40.15 | 116.28 |
| L168 | China | 40.15 | 116.28 |
| L156 | China | 48.29 | 128.08 |
| L097 | China | 40.15 | 116.28 |
| L160 | Canada | 43.4 | 79.25 |
| L161 | Canada | 43.4 | 79.25 |
| L162 | China | 40.145 | 116.275 |
| L145 | China | 40.85 | 122 |
| L165 | China | 40.85 | 122 |
| L062 | China | 40.85 | 122 |
| L060 | China | 40.85 | 122 |
| L114 | China | 40.85 | 122 |
| L012 | China | 40.85 | 122 |
| L065 | China | 40.85 | 122 |
| L063 | China | 40.85 | 122 |
| L170 | China | 40.85 | 122 |
| L059 | China | 40.85 | 122 |
| L146 | China | 40.85 | 122 |
| L164 | China | 40.85 | 122 |
| L123 | China | 48.29 | 128.08 |
| L064 | China | 40.85 | 122 |
| L144 | China | 40.85 | 122 |
| L061 | China | 40.85 | 122 |
| L058 | China | 40.85 | 122 |
| L077 | Italy | 41.54 | 12.3 |
| L117 | China | 48.29 | 128.08 |
| L108 | China | 48.29 | 128.08 |
| L099 | China | 40.145 | 116.275 |
| L093 | China | 39.19 | 116.285 |
| L084 | China | 40.145 | 116.275 |
| L070 | USA | 41.83 | 92.91 |
| L068 | USA | 41.83 | 92.91 |
| L081 | China | 43.35 | 126.285 |
| L067 | USA | 41.83 | 92.91 |
| L051 | China | 43.35 | 126.29 |
| L046 | China | 43.35 | 126.29 |
| L142 | China | 43.35 | 126.29 |
| L042 | China | 43.35 | 126.29 |
| L048 | China | 43.35 | 126.29 |
| L078 | Yugoslavia | 46.3 | 14.3 |
| L066 | China | 43.35 | 126.29 |
| L043 | China | 43.35 | 126.29 |
| L050 | China | 43.35 | 126.29 |
| L075 | Ukraine | 50.28 | 30.29 |
| L027 | China | 43.35 | 126.29 |
| L052 | China | 43.35 | 126.29 |
| L049 | China | 43.35 | 126.29 |
| L082 | China | 43.35 | 126.29 |
| L155 | China | 43.35 | 126.29 |
| L069 | America | 43.4 | 79.25 |
| L057 | China | 40.845 | 121.995 |
| L143 | China | 43.35 | 126.29 |
| L026 | China | 43.35 | 126.29 |
| L152 | China | 43.35 | 126.29 |
| L044 | China | 43.35 | 126.29 |
| L153 | Canada | 43.4 | 79.25 |
| L159 | Canada | 43.4 | 79.25 |
| L054 | Canada | 43.4 | 79.25 |
| L045 | China | 43.35 | 126.285 |
| L079 | Rumania | 44.23 | 26.1 |
| L071 | China | 44.49 | 111.7 |
| L072 | China | 44.49 | 111.7 |
| L073 | China | 44.49 | 111.7 |
| L148 | China | 44.49 | 111.7 |
| L147 | China | 44.49 | 111.7 |
| L039 | China | 48.29 | 128.08 |
| L030 | China | 48.29 | 128.08 |
| L017 | China | 48.29 | 128.08 |
| L109 | China | 48.29 | 128.08 |
| L005 | China | 48.29 | 128.08 |
| L021 | China | 48.29 | 128.08 |
| L115 | China | 48.29 | 128.08 |
| L276 | China | 40.845 | 121.995 |
| L105 | China | 48.29 | 128.08 |
| L129 | China | 48.29 | 128.08 |
| L033 | China | 48.29 | 128.08 |
| L013 | China | 48.29 | 128.08 |
| L112 | China | 48.29 | 128.08 |
| L020 | China | 48.29 | 128.08 |
| L025 | China | 48.29 | 128.08 |
| L003 | China | 48.29 | 128.08 |
| L110 | China | 48.29 | 128.08 |
| L001 | China | 48.29 | 128.08 |
| L173 | China | 48.29 | 128.08 |
| L120 | China | 48.29 | 128.08 |
| L111 | China | 48.29 | 128.08 |
| L138 | China | 48.29 | 128.08 |
| L022 | China | 48.29 | 128.08 |
| L130 | China | 48.29 | 128.08 |
| L278 | China | 44.49 | 111.695 |
| L041 | China | 48.29 | 128.08 |
| L107 | China | 48.29 | 128.08 |
| L006 | China | 48.29 | 128.08 |
| L011 | China | 48.29 | 128.08 |
| L038 | China | 48.29 | 128.08 |
| L034 | China | 48.29 | 128.08 |
| L047 | China | 43.35 | 126.285 |
| L141 | China | 48.29 | 128.08 |
| L035 | China | 48.29 | 128.08 |
| L135 | China | 48.29 | 128.08 |
| L007 | China | 48.29 | 128.08 |
| L158 | China | 48.29 | 128.08 |
| L103 | China | 48.29 | 128.08 |
| L037 | China | 48.29 | 128.08 |
| L132 | China | 48.29 | 128.08 |
| L104 | China | 48.29 | 128.08 |
| L014 | China | 48.29 | 128.08 |
| L016 | China | 48.29 | 128.08 |
| L131 | China | 48.29 | 128.08 |
| L134 | China | 48.29 | 128.08 |
| L004 | China | 48.29 | 128.08 |
| L040 | China | 48.29 | 128.08 |
| L151 | China | 48.29 | 128.08 |
| L008 | China | 48.29 | 128.08 |
| L113 | China | 48.29 | 128.08 |
| L032 | China | 48.29 | 128.08 |
| L157 | China | 48.29 | 128.08 |
| L140 | China | 48.29 | 128.08 |
| L024 | China | 48.29 | 128.08 |
| L127 | China | 48.29 | 128.08 |
| L167 | China | 48.29 | 128.08 |
| L166 | China | 48.29 | 128.08 |
| L080 | China | 48.29 | 128.08 |
| L126 | China | 48.29 | 128.08 |
| L128 | China | 48.29 | 128.08 |
| L009 | China | 48.29 | 128.08 |
| L133 | China | 48.29 | 128.08 |
| L150 | China | 48.29 | 128.08 |
| L137 | China | 48.29 | 128.08 |
| L102 | China | 48.29 | 128.08 |
| L018 | China | 48.29 | 128.08 |
| L175 | China | 48.29 | 128.08 |
| L031 | China | 48.29 | 128.08 |
| L119 | China | 48.29 | 128.08 |
| L029 | China | 48.29 | 128.08 |
| L087 | Germany | 52.31 | 13.2 |
| L088 | Russia | 61.5 | 98 |

**Table S2** Peak SNP associated with the resistance to the HG Type 0 and HG Type 1.2.3.5.7 identified by GWAS using the 3VmrMLM model single-environment method.

| SNP | Chr. | Position (bp) | Allele | HG Type | Environment | -LOG10(P) | r2(%) | significance | References |
| --- | --- | --- | --- | --- | --- | --- | --- | --- | --- |
| rs13363230 | 1 | 13363230 | G/T | race 4 | Harbin | 4.233874702 | 2.2309 | SUG | Yue et al. 2001 |
| rs27634423 | 1 | 27634423 | T/A | race 4 | Jilin | 5.374715419 | 3.4682 | SUG | Yue et al. 2001 |
|  |  |  |  | race 4 | Liaoning | 12.78240103 | 8.4174 | SIG | Yue et al. 2001 |
| rs35111254 | 1 | 35111254 | G/T | race 3 | Jilin | 7.829287052 | 5.5926 | SIG |  |
|  |  |  |  | race 3 | Liaoning | 9.782185141 | 6.757 | SIG |  |
| rs44155136 | 1 | 44155136 | C/T | race 4 | Harbin | 25.71995815 | 4.1155 | SIG |  |
| rs49760448 | 1 | 49760448 | C/A | race 4 | Harbin | 5.637945794 | 2.0772 | SUG | Wu et al. 2009 |
| rs51884258 | 1 | 51884258 | A/G | race 4 | Liaoning | 4.024592955 | 2.395 | SUG | Wu et al. 2009 |
| rs11916968 | 2 | 11916968 | C/A | race 4 | Harbin | 10.15039837 | 6.9767 | SIG |  |
| rs17071358 | 2 | 17071358 | C/A | race 4 | Liaoning | 6.88293967 | 3.8474 | SIG |  |
| rs48129464 | 2 | 48129464 | T/G | race 3 | Liaoning | 5.280733986 | 2.4767 | SUG |  |
| rs456656 | 3 | 456656 | T/C | race 4 | Jilin | 20.47366724 | 19.5574 | SIG |  |
|  |  |  |  | race 4 | Liaoning | 13.90300662 | 9.3462 | SIG |  |
|  |  |  |  | race 3 | Harbin | 16.35 | 15.6 | SIG |  |
| rs8515660 | 3 | 8515660 | A/G | race 3 | Liaoning | 4.78738442 | 2.4031 | SUG | Jiao et al. 2015A |
| rs19160692 | 4 | 19160692 | C/G | race 3 | Harbin | 30.51014712 | 6.2582 | SIG | Valdes-Lopez et al. 2011a |
| rs27757977 | 4 | 27757977 | G/T | race 3 | Harbin | 6.061772583 | 3.2578 | SIG | Valdes-Lopez et al. 2011a |
| rs29452411 | 4 | 29452411 | G/T | race 3 | Jilin | 5.503360632 | 4.2835 | SUG | Valdes-Lopez et al. 2011a |
| rs33309951 | 4 | 33309951 | C/A | race 3 | Jilin | 10.14668836 | 4.9686 | SIG | Valdes-Lopez et al. 2011a |
| rs43769905 | 4 | 43769905 | A/C | race 3 | Harbin | 4.769768935 | 4.5472 | SUG | Valdes-Lopez et al. 2011a |
| rs46410067 | 4 | 46410067 | A/G | race 3 | Jilin | 19.62125827 | 9.0298 | SIG | Valdes-Lopez et al. 2011a |
|  |  |  |  | race 3 | Liaoning | 20.09048066 | 7.7372 | SIG | Valdes-Lopez et al. 2011a |
| rs50885278 | 4 | 50885278 | A/G | race 4 | Jilin | 7.798927031 | 5.8871 | SIG |  |
|  |  |  |  | race 4 | Liaoning | 6.162662476 | 3.6043 | SIG |  |
| rs32462952 | 5 | 32462952 | G/A | race 3 | Jilin | 7.415523124 | 5.2512 | SIG | Yue et al. 2001 |
|  |  |  |  | race 3 | Liaoning | 9.99797881 | 5.8936 | SIG | Yue et al. 2001 |
| rs3265225 | 6 | 3265225 | G/T | race 4 | Harbin | 15.16945096 | 5.4989 | SIG |  |
| rs5726664 | 6 | 5726664 | G/T | race 3 | Jilin | 9.470874794 | 7.7936 | SIG |  |
|  |  |  |  | race 3 | Liaoning | 11.31226562 | 7.3874 | SIG |  |
| rs46720938 | 6 | 46720938 | C/T | race 4 | Harbin | 6.895588187 | 5.0512 | SIG |  |
| rs4028112 | 7 | 4028112 | A/T | race 4 | Harbin | 6.651443798 | 3.2738 | SIG |  |
| rs11642376 | 7 | 11642376 | C/A | race 4 | Jilin | 8.920481291 | 5.2053 | SIG |  |
| rs1809210 | 8 | 1809210 | G/T | race 3 | Harbin | 6.931115614 | 7.3681 | SIG |  |
| rs805006 | 8 | 805006 | C/A | race 3 | Harbin | 7.45 | 10.4 | SIG |  |
| rs22236673 | 9 | 22236673 | G/A | race 3 | Liaoning | 7.869201836 | 4.5058 | SIG | Wu et al. 2009 |
| rs33127400 | 9 | 33127400 | G/A | race 4 | Harbin | 36.3428469 | 6.9711 | SIG | Wu et al. 2009 |
| rs1639202 | 10 | 1639202 | C/A | race 3 | Harbin | 9.345005615 | 11.5859 | SIG |  |
| rs6241182 | 11 | 6241182 | C/A | race 3 | Harbin | 15.1663522 | 8.3577 | SIG |  |
| rs804370 | 13 | 804370 | C/T | race 3 | Jilin | 4.040259075 | 2.9855 | SUG |  |
| rs1433972 | 13 | 1433972 | C/A | race 3 | Liaoning | 9.427095731 | 1.7186 | SIG |  |
| rs6717162 | 13 | 6717162 | C/A | race 4 | Harbin | 5.853382204 | 4.1803 | SIG |  |
| rs15274949 | 13 | 15274949 | A/G | race 3 | Liaoning | 11.97119859 | 8.0165 | SIG |  |
| rs22938480 | 13 | 22938480 | G/A | race 4 | Harbin | 4.495098686 | 3.1933 | SUG | Valdes-Lopez et al. 2011a |
| rs23114690 | 13 | 23114690 | G/T | race 4 | Jilin | 7.769746676 | 5.2505 | SIG | Valdes-Lopez et al. 2011a |
| rs23148030 | 13 | 23148030 | G/T | race 4 | Liaoning | 6.534465041 | 3.6106 | SIG | Valdes-Lopez et al. 2011a |
| rs24026375 | 13 | 24026375 | G/T | race 4 | Jilin | 8.373451852 | 6.4348 | SIG |  |
|  |  |  |  | race 4 | Liaoning | 7.397039816 | 4.6667 | SIG |  |
| rs15049524 | 14 | 15049524 | C/T | race 4 | Jilin | 12.22976965 | 10.3712 | SIG |  |
|  |  |  |  | race 4 | Liaoning | 12.57969391 | 8.441 | SIG |  |
| rs17861986 | 14 | 17861986 | C/A | race 3 | Harbin | 8.606866495 | 5.5587 | SIG |  |
| rs18628696 | 14 | 18628696 | C/A | race 3 | Jilin | 21.19107064 | 3.0741 | SIG |  |
|  |  |  |  | race 3 | Liaoning | 28.52065381 | 4.0512 | SIG |  |
| rs22586702 | 14 | 22586702 | G/T | race 4 | Liaoning | 6.040995322 | 3.7296 | SIG |  |
| rs35020134 | 14 | 35020134 | G/A | race 4 | Jilin | 13.43544884 | 2.2107 | SIG |  |
| rs11720920 | 15 | 11720920 | C/A | race 4 | Jilin | 22.03505536 | 3.9921 | SIG | Kabelka et al. 2005 |
|  |  |  |  | race 4 | Liaoning | 26.56943573 | 4.1231 | SIG | Kabelka et al. 2005 |
| rs13361457 | 15 | 13361457 | A/C | race 4 | Harbin | 9.203658172 | 6.894 | SIG | Kabelka et al. 2005 |
| rs32951277 | 15 | 32951277 | T/C | race 3 | Jilin | 5.237774909 | 4.1162 | SUG | Kabelka et al. 2005 |
| rs33704130 | 16 | 33704130 | A/C | race 3 | Harbin | 8.41 | 7.65 | SIG |  |
|  |  |  |  | race 4 | Harbin | 6.7 | 7.74 | SIG |  |
| rs8342534 | 17 | 8342534 | T/C | race 4 | Harbin | 26.1806168 | 3.6446 | SIG |  |
| rs27444613 | 17 | 27444613 | T/G | race 3 | Harbin | 8.760151502 | 10.7145 | SIG | Kazi et al. 2010 |
| rs38535689 | 17 | 38535689 | G/T | race 3 | Liaoning | 10.73681065 | 3.4731 | SIG |  |
| rs47402736 | 18 | 47402736 | T/A | race 3 | Jilin | 13.22463402 | 11.759 | SIG | Winter et al. 2007 |
|  |  |  |  | race 3 | Liaoning | 9.351008329 | 6.451 | SIG | Winter et al. 2007 |
| rs24929103 | 20 | 24929103 | C/T | race 3 | Jilin | 4.898678866 | 3.3795 | SUG | Qiu et al. 1999 |
| rs47494683 | 20 | 47494683 | C/T | race 4 | Liaoning | 12.84674443 | 3.0808 | SIG | Jiao et al. 2015A |

**Table S3** Peak SNP associated with the resistance to the HG Type 0 and HG Type 1.2.3.5.7 identified by GWAS using the 3VmrMLM model multiple-environment method.

| SNP | Chr. | Position (bp) | Allele | HG Type | -LOG10(P) | r2(%) | significance | References |
| --- | --- | --- | --- | --- | --- | --- | --- | --- |
| rs27634423 | 1 | 27634423 | T/A | race 4 | 8.573857758 | 1.7176 | SIG |  |
| rs35111254 | 1 | 35111254 | G/T | race 3 | 10.47147187 | 3.0638 | SIG |  |
| rs39079225 | 1 | 39079225 | C/A | race 4 | 13.7874902 | 2.8742 | SIG |  |
| rs3875620 | 2 | 3875620 | C/T | race 4 | 5.586414913 | 1.1132 | SUG |  |
| rs17071358 | 2 | 17071358 | C/A | race 4 | 9.406455936 | 1.6479 | SIG |  |
| rs456656 | 3 | 456656 | T/C | race 4 | 33.79189902 | 8.0233 | SIG |  |
| rs13078029 | 3 | 13078029 | A/G | race 3 | 11.63866851 | 1.7684 | SIG | Jiao et al. 2015A |
| rs3117588 | 4 | 3117588 | C/T | race 3 | 33.35709785 | 2.0805 | SIG | Vuong et al. 2011 |
| rs6178757 | 4 | 6178757 | G/T | race 4 | 11.86296662 | 0.66 | SIG | Wu et al. 2009 |
| rs40146893 | 4 | 40146893 | T/G | race 3 | 37.75820266 | 8.6073 | SIG | Valdes-Lopez et al. 2011a |
| rs46410067 | 4 | 46410067 | A/G | race 3 | 17.87182633 | 2.5986 | SIG | Valdes-Lopez et al. 2011a |
| rs48683585 | 4 | 48683585 | A/G | race 4 | 9.999147969 | 1.7545 | SIG |  |
| rs50885278 | 4 | 50885278 | A/G | race 4 | 4.18543368 | 0.827 | SUG |  |
| rs18311998 | 5 | 18311998 | G/T | race 3 | 11.18524167 | 1.1822 | SIG | Yue et al. 2001 |
| rs21843247 | 5 | 21843247 | T/A | race 3 | 8.529926747 | 0.9942 | SIG | Yue et al. 2001 |
| rs32462952 | 5 | 32462952 | G/A | race 3 | 8.114855718 | 1.3517 | SIG | Yue et al. 2001 |
| rs5441402 | 6 | 5441402 | G/T | race 3 | 16.70248853 | 1.4848 | SIG |  |
| rs5726664 | 6 | 5726664 | G/T | race 3 | 14.75865408 | 4.3645 | SIG |  |
| rs13550557 | 6 | 13550557 | T/A | race 3 | 10.73315579 | 2.1029 | SIG | Mahalingam 1995 |
| rs1747996 | 7 | 1747996 | G/T | race 4 | 23.04287479 | 1.5243 | SIG |  |
| rs13199425 | 8 | 13199425 | C/T | race 4 | 12.29199824 | 1 | SIG | Mahalingam 1995 |
| rs22236673 | 9 | 22236673 | G/A | race 3 | 4.825021146 | 1.393 | SUG | Wu et al. 2009 |
| rs42625306 | 10 | 42625306 | C/A | race 4 | 11.06984657 | 1.4414 | SIG | Vuong et al. 2010 |
| rs15274949 | 13 | 15274949 | A/G | race 3 | 3.636874623 | 0.9425 | SUG |  |
| rs23148030 | 13 | 23148030 | G/T | race 4 | 15.6361376 | 3.4247 | SIG | Valdes-Lopez et al. 2011a |
| rs24026375 | 13 | 24026375 | G/T | race 4 | 27.20264692 | 2.9766 | SIG | Valdes-Lopez et al. 2011a |
| rs15049524 | 14 | 15049524 | C/T | race 4 | 7.650568005 | 1.5644 | SIG |  |
| rs22586702 | 14 | 22586702 | G/T | race 4 | 18.75686438 | 4.1102 | SIG |  |
| rs6636391 | 15 | 6636391 | G/T | race 4 | 19.5771446 | 1.6942 | SIG | Wang et al. 2001 |
| rs20183947 | 17 | 20183947 | C/T | race 4 | 34.81461674 | 1.586 | SIG | Kazi et al. 2010 |
| rs37703714 | 17 | 37703714 | G/A | race 3 | 5.988093848 | 1.7338 | SIG |  |
| rs9528564 | 18 | 9528564 | A/G | race 3 | 8.791243206 | 2.4794 | SIG |  |
| rs21379503 | 18 | 21379503 | G/T | race 4 | 10.36804712 | 1.2407 | SIG |  |
| rs47402736 | 18 | 47402736 | T/A | race 3 | 15.075767 | 4.5547 | SIG | Winter et al. 2007 |
| rs42627536 | 19 | 42627536 | T/C | race 3 | 13.79157989 | 2.571 | SIG | Guo et al. 2006 |
| rs2027360 | 20 | 2027360 | C/T | race 4 | 7.876292375 | 1.1849 | SIG |  |

**Table S4** Peak SNP associated with the resistance to the HG Type 0 and HG Type 1.2.3.5.7 identified by GWAS using QEI detection of 3VmrMLM multiple-environment method.

| SNP | Chr. | Position (bp) | Allele | HG Type | -LOG10(P) | r2(%) | significance | References |
| --- | --- | --- | --- | --- | --- | --- | --- | --- |
| rs51884258 | 1 | 51884258 | A/G | race 4 | 8.29998987 | 2.0728 | SIG | Wu et al. 2009 |
| rs456656 | 3 | 456656 | T/C | race 4 | 5.585216385 | 1.439 | SUG |  |
| rs28225475 | 4 | 28225475 | G/C | race 3 | 6.663975314 | 2.3358 | SIG | Valdes-Lopez et al. 2011a |
| rs43537009 | 9 | 43537009 | A/G | race 4 | 6.538126659 | 1.6608 | SIG | Winter et al. 2007 |
| rs14265606 | 13 | 14265606 | A/T | race 3 | 23.62119333 | 8.1497 | SIG |  |
| rs15049524 | 14 | 15049524 | C/T | race 4 | 19.75798756 | 4.8839 | SIG |  |
| rs48079067 | 18 | 48079067 | G/T | race 3 | 6.99221568 | 2.4416 | SIG | Winter et al. 2007 |
| rs50037674 | 18 | 50037674 | G/T | race 3 | 13.44663415 | 4.565 | SIG | Winter et al. 2007 |
| rs34900979 | 20 | 34900979 | C/T | race 4 | 48.82092457 | 12.810 | SIG | Qiu et al. 1999 |

**Table S5** Significant SNPs and predicted candidate genes associated with SCN HG Type 0 and HG Type 1.2.3.5.7 resistance in soybean

| Peak SNP | Chr. | Physical position (bp) | Gene ID | Start Position | Stop Position | Functional annotation |
| --- | --- | --- | --- | --- | --- | --- |
| rs35111254 | 1 | 35111254 | Glyma.01G104000 | 35075339 | 35076201 |  |
|  |  |  | Glyma.01G104100 | 35076719 | 35085254 | isochorismate synthase 2 |
|  |  |  | Glyma.01G104200 | 35104237 | 35111049 | POX (plant homeobox) family protein |
|  |  |  | Glyma.01G104300 | 35134820 | 35135780 |  |
| rs17071358 | 2 | 17071358 | Glyma.02G157300 | 17013268 | 17014742 | RAD-like 1 |
|  |  |  | Glyma.02G157400 | 17027118 | 17028035 |  |
|  |  |  | Glyma.02G157500 | 17030537 | 17031115 |  |
|  |  |  | Glyma.02G157600 | 17056138 | 17056482 | BED zinc finger ;hAT family dimerisation domain |
|  |  |  | Glyma.02G157700 | 17060683 | 17071744 | binding |
|  |  |  | Glyma.02G157800 | 17128244 | 17133221 | Transcription elongation factor (TFIIS) family protein |
|  |  |  | Glyma.02G157900 | 17155099 | 17156564 |  |
| rs456656 | 3 | 456656 | Glyma.03G004000 | 357269 | 360639 | alpha-L-arabinofuranosidase 1 |
|  |  |  | Glyma.03G004100 | 364853 | 366774 | calmodulin 5 |
|  |  |  | Glyma.03G004200 | 370538 | 374055 | ubiquitin-conjugating enzyme 22 |
|  |  |  | Glyma.03G004300 | 375732 | 377625 |  |
|  |  |  | Glyma.03G004400 | 377626 | 383458 | proteasome family protein |
|  |  |  | Glyma.03G004500 | 391913 | 396356 | Protein kinase superfamily protein |
|  |  |  | Glyma.03G004600 | 398842 | 404874 | Sec14p-like phosphatidylinositol transfer family protein |
|  |  |  | Glyma.03G004700 | 406773 | 408434 | Protein of unknown function (DUF1138) |
|  |  |  | Glyma.03G004800 | 418391 | 423868 |  |
|  |  |  | Glyma.03G004900 | 426042 | 442555 | ENTH/VHS family protein |
|  |  |  | Glyma.03G005000 | 444058 | 450572 | ELMO/CED-12 family protein |
|  |  |  | Glyma.03G005100 | 452956 | 454508 |  |
|  |  |  | Glyma.03G005200 | 459177 | 462834 | MATE efflux family protein |
|  |  |  | Glyma.03G005300 | 465177 | 470215 | MATE efflux family protein |
|  |  |  | Glyma.03G005400 | 483748 | 490996 | MATE efflux family protein |
|  |  |  | Glyma.03G005500 | 495973 | 501101 | MATE efflux family protein |
|  |  |  | Glyma.03G005600 | 504869 | 509004 | MATE efflux family protein |
|  |  |  | Glyma.03G005700 | 521423 | 528409 | methylthioalkylmalate synthase-like 4 |
|  |  |  | Glyma.03G005800 | 545102 | 549977 | MATE efflux family protein |
|  |  |  | Glyma.03G005900 | 556124 | 561374 | Co-chaperone GrpE family protein |
| rs50885278 | 4 | 50885278 | Glyma.04G239400 | 50786868 | 50788346 | MAP kinase substrate 1 |
|  |  |  | Glyma.04G239500 | 50798520 | 50799807 | Dof-type zinc finger DNA-binding family protein |
|  |  |  | Glyma.04G239600 | 50807696 | 50811314 | Nuclear transport factor 2 (NTF2) family protein |
|  |  |  | Glyma.04G239700 | 50817842 | 50822050 | Leucine-rich repeat transmembrane protein kinase family protein |
|  |  |  | Glyma.04G239800 | 50826167 | 50829145 | Thioredoxin superfamily protein |
|  |  |  | Glyma.04G239900 | 50835924 | 50838342 | glycolipid transfer protein 2 |
|  |  |  | Glyma.04G240000 | 50847794 | 50850747 | nodulin MtN21 /EamA-like transporter family protein |
|  |  |  | Glyma.04G240100 | 50852103 | 50864161 | nodulin MtN21 /EamA-like transporter family protein |
|  |  |  | Glyma.04G240200 | 50853401 | 50854079 | nodulin MtN21 /EamA-like transporter family protein |
|  |  |  | Glyma.04G240300 | 50865799 | 50871027 | Protein of unknown function (DUF300) |
|  |  |  | Glyma.04G240400 | 50873113 | 50880698 | vacuolar protein sorting 45 |
|  |  |  | Glyma.04G240500 | 50882258 | 50885037 | Uncharacterised protein family UPF0090 |
|  |  |  | Glyma.04G240600 | 50888492 | 50892907 | serine carboxypeptidase-like 42 |
|  |  |  | Glyma.04G240700 | 50902394 | 50905765 | TRICHOME BIREFRINGENCE-LIKE 7 |
|  |  |  | Glyma.04G240800 | 50907553 | 50910551 | alcohol dehydrogenase 1 |
|  |  |  | Glyma.04G240900 | 50913301 | 50922892 | global transcription factor group A2 |
|  |  |  | Glyma.04G241000 | 50925586 | 50927969 | Mog1/PsbP/DUF1795-like photosystem II reaction center PsbP family protein |
|  |  |  | Glyma.04G241100 | 50928362 | 50929413 | Pentatricopeptide repeat (PPR-like) superfamily protein |
|  |  |  | Glyma.04G241200 | 50932741 | 50934318 |  |
|  |  |  | Glyma.04G241300 | 50933661 | 50934017 |  |
|  |  |  | Glyma.04G241400 | 50942480 | 50947171 | Nodulin MtN3 family protein |
|  |  |  | Glyma.04G241500 | 50954367 | 50955220 |  |
|  |  |  | Glyma.04G241600 | 50962156 | 50962843 |  |
|  |  |  | Glyma.04G241700 | 50968365 | 50969124 |  |
|  |  |  | Glyma.04G241800 | 50972803 | 50977112 |  |
| rs5726664 | 6 | 5726664 | Glyma.06G073000 | 5626756 | 5630100 | Protein phosphatase 2A regulatory B subunit family protein |
|  |  |  | Glyma.06G073100 | 5634772 | 5639307 | RING/U-box superfamily protein |
|  |  |  | Glyma.06G073200 | 5644140 | 5650953 | SPOC domain / Transcription elongation factor S-II protein |
|  |  |  | Glyma.06G073300 | 5651331 | 5654202 | DNAJ heat shock family protein |
|  |  |  | Glyma.06G073400 | 5655770 | 5657742 | CTC-interacting domain 5 |
|  |  |  | Glyma.06G073500 | 5659171 | 5661096 |  |
|  |  |  | Glyma.06G073600 | 5664129 | 5669127 | Mog1/PsbP/DUF1795-like photosystem II reaction center PsbP family protein |
|  |  |  | Glyma.06G073700 | 5670056 | 5689370 | embryo defective 2410 |
|  |  |  | Glyma.06G073800 | 5691727 | 5694562 |  |
|  |  |  | Glyma.06G073900 | 5696712 | 5700728 | syntaxin of plants 32 |
|  |  |  | Glyma.06G074000 | 5701847 | 5704926 | syntaxin of plants 32 |
|  |  |  | Glyma.06G074100 | 5710970 | 5712358 | molybdate transporter 1 |
|  |  |  | Glyma.06G074200 | 5715485 | 5718978 | SWIM zinc finger family protein |
|  |  |  | Glyma.06G074300 | 5724246 | 5730063 | CHY-type/CTCHY-type/RING-type Zinc finger protein |
|  |  |  | Glyma.06G074400 | 5732302 | 5735942 | Protein of unknown function (DUF581) |
|  |  |  | Glyma.06G074500 | 5750390 | 5754119 |  |
|  |  |  | Glyma.06G074600 | 5751257 | 5757542 | potassium channel in Arabidopsis thaliana 3 |
|  |  |  | Glyma.06G074700 | 5758402 | 5786537 |  |
|  |  |  | Glyma.06G074800 | 5788556 | 5792829 |  |
|  |  |  | Glyma.06G074900 | 5793880 | 5795448 | N-acetyl-l-glutamate kinase |
|  |  |  | Glyma.06G075000 | 5796960 | 5805987 | DDB1-CUL4 associated factor 1 |
|  |  |  | Glyma.06G075100 | 5807768 | 5812501 | O-Glycosyl hydrolases family 17 protein |
|  |  |  | Glyma.06G075200 | 5816893 | 5821265 | Protein of unknown function (DUF1218) |
|  |  |  | Glyma.06G075300 | 5822401 | 5833249 | SHK1 binding protein 1 |
|  |  |  | Glyma.06G075400 | 5824447 | 5825012 |  |
| rs14265606 | 13 | 14265606 | Glyma.13G047000 | 14170795 | 14171893 |  |
|  |  |  | Glyma.13G047100 | 14172381 | 14179987 |  |
|  |  |  | Glyma.13G047200 | 14190107 | 14191092 |  |
|  |  |  | Glyma.13G047300 | 14193855 | 14196435 | allene oxide cyclase 3 |
|  |  |  | Glyma.13G047400 | 14255642 | 14257802 | TCP family transcription factor |
|  |  |  | Glyma.13G047500 | 14291927 | 14296667 | NagB/RpiA/CoA transferase-like superfamily protein |
|  |  |  | Glyma.13G047600 | 14298572 | 14300040 | cytochrome B561-1 |
|  |  |  | Glyma.13G047700 | 14303335 | 14313128 | Proline-rich spliceosome-associated (PSP) family protein / zinc knuckle (CCHC-type) family protein |
|  |  |  | Glyma.13G047800 | 14323678 | 14328861 | gamete-expressed 3 |
|  |  |  | Glyma.13G047900 | 14333624 | 14339258 |  |
| rs15274949 | 13 | 15274949 | Glyma.13G054500 | 15173682 | 15182007 | ARM repeat superfamily protein |
|  |  |  | Glyma.13G054600 | 15191769 | 15194310 | Pectin lyase-like superfamily protein |
|  |  |  | Glyma.13G054700 | 15208076 | 15209264 |  |
|  |  |  | Glyma.13G054800 | 15215629 | 15217477 |  |
|  |  |  | Glyma.13G054900 | 15221732 | 15222281 |  |
|  |  |  | Glyma.13G055000 | 15224497 | 15225629 |  |
|  |  |  | Glyma.13G055100 | 15228485 | 15228973 |  |
|  |  |  | Glyma.13G055200 | 15229290 | 15234025 | Proteasome component (PCI) domain protein |
|  |  |  | Glyma.13G055300 | 15237358 | 15240608 | O-fucosyltransferase family protein |
|  |  |  | Glyma.13G055400 | 15248277 | 15250317 | SPIRAL1-like1 |
|  |  |  | Glyma.13G055500 | 15288951 | 15291189 | arogenate dehydrogenase |
|  |  |  | Glyma.13G055600 | 15309709 | 15313747 | Haloacid dehalogenase-like hydrolase (HAD) superfamily protein |
|  |  |  | Glyma.13G055700 | 15329124 | 15335727 |  |
|  |  |  | Glyma.13G055800 | 15336924 | 15337076 |  |
|  |  |  | Glyma.13G055900 | 15339154 | 15345466 |  |
|  |  |  | Glyma.13G056000 | 15348689 | 15350115 |  |
| rs24026375 | 13 | 24026375 | Glyma.13G126000 | 23922920 | 23927833 | cellulose synthase A4 |
|  |  |  | Glyma.13G126100 | 23945123 | 23945287 |  |
|  |  |  | Glyma.13G126200 | 23951477 | 23958210 | phosphate starvation response 1 |
|  |  |  | Glyma.13G126300 | 23969964 | 23971099 |  |
|  |  |  | Glyma.13G126400 | 23974970 | 23976380 | Ribosomal protein L14p/L23e family protein |
|  |  |  | Glyma.13G126500 | 23978122 | 23979209 | Glutathione S-transferase family protein |
|  |  |  | Glyma.13G126600 | 23979724 | 23986655 | SET-domain containing protein lysine methyltransferase family protein |
|  |  |  | Glyma.13G126700 | 23992461 | 23994040 | plasmodesmata-located protein 2 |
|  |  |  | Glyma.13G126800 | 23999717 | 23999869 |  |
|  |  |  | Glyma.13G126900 | 24000389 | 24001338 | translocase of outer membrane 22-V |
|  |  |  | Glyma.13G127000 | 24008533 | 24012615 | indole-3-acetic acid inducible 11 |
|  |  |  | Glyma.13G127100 | 24013027 | 24016923 | Leucine-rich repeat transmembrane protein kinase family protein |
|  |  |  | Glyma.13G127200 | 24024826 | 24026790 | photosystem II reaction center PSB28 protein |
|  |  |  | Glyma.13G127300 | 24028455 | 24031330 | Exostosin family protein |
|  |  |  | Glyma.13G127400 | 24032157 | 24038754 | NAD(P)-binding Rossmann-fold superfamily protein |
|  |  |  | Glyma.13G127500 | 24041356 | 24050612 | multidrug resistance-associated protein 5 |
|  |  |  | Glyma.13G127600 | 24069639 | 24073819 | RPM1 interacting protein 13 |
|  |  |  | Glyma.13G127700 | 24076156 | 24077910 | hydroxyproline-rich glycoprotein family protein |
|  |  |  | Glyma.13G127800 | 24080737 | 24086760 | Tetratricopeptide repeat (TPR)-like superfamily protein |
|  |  |  | Glyma.13G127900 | 24087878 | 24089432 | GroES-like zinc-binding dehydrogenase family protein |
|  |  |  | Glyma.13G128000 | 24090049 | 24095149 | Beta-ketoacyl synthase |
|  |  |  | Glyma.13G128100 | 24098615 | 24099676 | RmlC-like cupins superfamily protein |
|  |  |  | Glyma.13G128200 | 24104441 | 24112813 | Protein kinase superfamily protein |
|  |  |  | Glyma.13G128300 | 24122930 | 24129635 | pfkB-like carbohydrate kinase family protein |
| rs18628696 | 14 | 18628696 | Glyma.14G123600 | 18537222 | 18537513 |  |
|  |  |  | Glyma.14G123700 | 18589117 | 18599795 | plastid division1 |
|  |  |  | Glyma.14G123800 | 18634015 | 18635321 |  |
| rs22586702 | 14 | 22586702 | Glyma.14G131400 | 22505022 | 22506112 |  |
|  |  |  | Glyma.14G131500 | 22508168 | 22508443 |  |
|  |  |  | Glyma.14G131600 | 22543570 | 22545455 |  |
|  |  |  | Glyma.14G131700 | 22555716 | 22558927 | Protein of unknown function (DUF1218) |
|  |  |  | Glyma.14G131800 | 22561942 | 22562718 |  |
|  |  |  | Glyma.14G131900 | 22580087 | 22586928 |  |
|  |  |  | Glyma.14G132000 | 22609490 | 22618264 | Protein of unknown function (DUF1997) |
|  |  |  | Glyma.14G132100 | 22626936 | 22629212 | maturase K |
|  |  |  | Glyma.14G132200 | 22632092 | 22632391 | NADH-ubiquinone oxidoreductase B18 subunit, putative |
|  |  |  | Glyma.14G132300 | 22684278 | 22688050 | mraW methylase family protein |
| rs15049524 | 14 | 15049524 | none | none | none |  |

| **Table S6** Primers used for quantitative real-time PCR. | | |
| --- | --- | --- |
| Gene ID | Forward primer (5'-3') | Reverse primer (5'-3') |
| *Glyma.03G004300* | CATATGCGATGTTGCCTACAAA | GTCAAATGCAGATGTGAGATCC |
| *Glyma.03G005600* | TTTCACTTGCCAGAATTTCCTG | CAGGAATCTCGAGCTTGAATTG |
| *Glyma.04G239400* | GTGTTTGTGCTCTCGAATTTCT | ATGATCAAAGGTTGCCAGAGTA |
| *Glyma.04G240300* | CCAAGCAGAAAGAGACAACATC | TTATCAGCCCTGTACATGCTAG |
| *Glyma.04G240800* | GCTGCTGTTATTGTCTAATGCA | GACATGCGTTGAACAAATTGTG |
| *Glyma.04G241400* | TCTCTGTTTGTTTTTCTCTGCG | CTGTAACGCCTTTATTGTCTCG |
| *Glyma.06G074700* | CTCCTTGTTCTTTCCTCCTGAT | CACAATAGAGGCAAATACGAGC |
| *Glyma.13G127100* | CAAGTACAATTGTGGCAGTCAA | ACTATCATCACATCAGCGTCAT |
| *Glyma.13G128200* | CCATCAAAGCTCATTCACTCAC | GAAGAAGAAGAAAACCACACCG |
| *Glyma.13G128300* | GAGCTTGTTGATACAACTGGTG | TGAAGCCTTGTTTCTTGCATAC |
| *GmActin4* | GTTTCAAGCTCTTGCTCGTAATCA | GTGTCAGCCATACTGTCCCCATTT |
